# Identification of osmoadaptive strategies in the halophile, heterotrophic ciliate *Schmidingerothrix salinarum*

**DOI:** 10.1371/journal.pbio.2003892

**Published:** 2018-01-22

**Authors:** Lea Weinisch, Steffen Kühner, Robin Roth, Maria Grimm, Tamara Roth, Daili J. A. Netz, Antonio J. Pierik, Sabine Filker

**Affiliations:** 1 Department of Molecular Ecology, University of Kaiserslautern, Kaiserslautern, Germany; 2 Department of Ecology, University of Kaiserslautern, Kaiserslautern, Germany; 3 Department of Biochemistry, University of Kaiserslautern, Kaiserslautern, Germany; Max Planck Institute for Terrestrial Microbiology, Germany

## Abstract

Hypersaline environments pose major challenges to their microbial residents. Microorganisms have to cope with increased osmotic pressure and low water activity and therefore require specific adaptation mechanisms. Although mechanisms have already been thoroughly investigated in the green alga *Dunaliella salina* and some halophilic yeasts, strategies for osmoadaptation in other protistan groups (especially heterotrophs) are neither as well known nor as deeply investigated as for their prokaryotic counterpart. This is not only due to the recent awareness of the high protistan diversity and ecological relevance in hypersaline systems, but also due to methodological shortcomings. We provide the first experimental study on haloadaptation in heterotrophic microeukaryotes, using the halophilic ciliate *Schmidingerothrix salinarum* as a model organism. We established three approaches to investigate fundamental adaptation strategies known from prokaryotes. First, proton nuclear magnetic resonance (^1^H-NMR) spectroscopy was used for the detection, identification, and quantification of intracellular compatible solutes. Second, ion-imaging with cation-specific fluorescent dyes was employed to analyze changes in the relative ion concentrations in intact cells. Third, the effect of salt concentrations on the catalytic performance of *S*. *salinarum* malate dehydrogenase (MDH) and isocitrate dehydrogenase (ICDH) was determined. ^1^H-NMR spectroscopy identified glycine betaine (GB) and ectoine (Ect) as the main compatible solutes in *S*. *salinarum*. Moreover, a significant positive correlation of intracellular GB and Ect concentrations and external salinity was observed. The addition of exogenous GB, Ect, and choline (Ch) stimulated the cell growth notably, indicating that *S*. *salinarum* accumulates the solutes from the external medium. Addition of external ^13^C_2_-Ch resulted in conversion to ^13^C_2_-GB, indicating biosynthesis of GB from Ch. An increase of external salinity up to 21% did not result in an increase in cytoplasmic sodium concentration in *S*. *salinarum*. This, together with the decrease in the catalytic activities of MDH and ICDH at high salt concentration, demonstrates that *S*. *salinarum* employs the salt-out strategy for haloadaptation.

## Author summary

Salinity is one of the strongest abiotic factors in nature and can have harmful effects on organisms. When a cell is placed in a high-salt environment, osmotic pressure pulls unbound water molecules out of the cell, leaving behind a highly concentrated cytoplasm, which impairs cell function and can cause cell death. Organisms living in high-salt environments therefore need to have specific adaptation mechanisms. For prokaryotes and fungi, such adaptations have been examined in detail. Protists—unicellular eukaryotes that constitute most of the eukaryotic diversity—are less well studied, mainly because of the lack of adequate methodological approaches. The scope of our study therefore includes the establishment of techniques to investigate fundamental salt adaptation strategies in protists and the application of these techniques to a halophilic, heterotroph model ciliate protist to reveal its specific haloadaptations. We provide the first experimental evidence for the biosynthesis and accumulation of two compatible solutes that the ciliate uses to combat increasing salt concentrations. One of these, glycine betaine, is widely distributed across kingdoms, while the other, ectoine, has to date only been identified in prokaryotes.

## Introduction

Salinity is a decisive environmental determinant of microbial community composition and dispersal, exerting a high evolutionary selection pressure [1]. Shifts in salinity represent one of the most difficult environmental barriers to cross for macro- [2,3] and microorganisms alike [4,5]. Reasons are mainly the energetic costs of osmoregulation and the requirement of specific adaptive mechanisms to cope with high salt concentrations [6]. Salinity has detrimental effects on organisms when not counteracted. Hypertonic conditions induce an osmotic gradient, which lowers the relative water content of a cell, leaving behind a highly concentrated cytoplasm. In such a cell environment, nucleic acids, proteins, and other macromolecules denature and lose their functions [7]. Proteins form aggregates, precipitate [8], and disrupt their tertiary structure. Enzymes lose their flexibility and, consequently, their catalytic activity [9]. Nevertheless, flourishing communities of archaea, bacteria, and microbial eukaryotes are found in various hypersaline environments on Earth [10].

Due to the only recent awareness of the ecological importance of microbial eukaryotes in hypersaline systems [11,12], most knowledge about haloadaptation strategies derives from prokaryotic research, in which in-depth investigations already revealed a broad repertoire of different haloadaptation mechanisms. We, here, describe the two main and fundamentally different processes as adaptations to high-salt environments (reviewed in detail by [13]). The salt-in strategy involves the intracellular accumulation of molar concentrations of chloride ions (Cl^−^) and potassium ions (K^+^). Because proteins need to retain their functional conformation and activity at high intracellular salt concentrations, massive adaptations of the enzymatic machinery are mandatory [14]. Salt-in strategists, therefore, frequently possess highly acidic proteomes. Additionally, halophilic proteins are enriched with hydrophilic amino acids [14], which prevent overly rigidly folded conformations under high-salt conditions. Consequently, hypoosmotic conditions cannot easily be counterbalanced and lead to destabilization and malfunction of proteins. Thus, the salt-in strategy is used by few groups of halophiles only, such as the aerobic members of Halobacteriaceae (Archaea), *Salinibacter* (Bacteria), and the anaerobic Halanaerobiales (Bacteria). Alternatively, the more common salt-out strategy involves the exclusion of salt ions from the cytoplasm, simultaneously synthesizing or accumulating high concentrations of compatible solutes. These are uncharged or zwitterionic low molecular mass organic compounds, which do not interfere with the cell metabolism at high cytoplasmatic concentrations, provide osmotic balance, and further protect (nonsalt-adapted) enzymes and other macromolecular structures against inactivation, inhibition, and denaturation [15].

Knowledge about microeukaryotic salt adaptation strategies is restricted to some genome-sequenced fungi (halotolerant *Hortea werneckii*, halophilic *Wallemia ichtyophaga*, and halophilic *Eurotium rubrum*), and the green alga *Dunaliella*. Even though all three fungal taxa share some common haloadaptive traits, the differences in their genetic repertoire suggest multiple molecular pathways for haloadaptation and a high diversity in adaptive strategies [16]. All three fungi accumulate glycerol as their main compatible solute [16–18] and have a significantly higher proportion of acidic residues in predicted proteins [16]. Genome and transcriptome analyses of *H*. *werneckii*, which maintains low intracellular K^+^ and sodium ion (Na^+^) concentrations, revealed considerable expansion of gene families encoding membrane-bound metal cation transporters [17,19,20]. Interestingly, the halophiles *W*. *ichthyophaga* and *E*. *rubrum* have a substantially lower number of such genes [18–20]. This indicates that in contrast to *H*. *werneckii*, elevated intracellular Na^+^ concentrations are not toxic for *W*. *ichthyophaga*, further documenting the different salt-combatting strategies in these two model fungi. Intracellular accumulation of Na^+^ to promote growth under salt stress was also reported for the halotolerant yeast *Debaryomyces hansenii* [21]. Transcriptome data of *D*. *salina* identified an active signaling system, which enables it to quickly adapt to a wide variety of salt concentrations as well as a significantly enriched photosynthetic glycerol pathway as adaptation to osmotic changes [22]. This corroborates earlier findings of low intracellular Na^+^-concentrations [23] and an electron transport-coupled Na^+^-extrusion [24] in *D*. *salina*. Only recently Harding et al. [25] published a transcriptome study of the halophilic flagellates *Halocafeteria seosinensis* and *Pharyngomonas kirbyi* to investigate salt adaptation strategies in heterotroph protists. Both species do not possess an acidic proteome characteristic for salt-in strategists, but display an increased expression of enzymes involved in the synthesis and transport of the compatible solutes 5-hydroxyectoine and myo-inositol.

However, up to now there is no experimental evidence for intracellular ion concentrations and the accumulation and synthesis of compatible solutes with increasing salinity in heterotrophic protists. This is mainly due to methodological reasons, such as the lack of axenic cultures, disturbance of measurements of intracellular contents by ingested food bacteria, or the lack of adequate sensitive techniques. Methods, which measure intracellular concentrations of inorganic ions and other compounds, were so far mainly applied to bacteria (e.g., [26–29]). These methods rely on the measurement of intracellular and extracellular water volumes, which are used to determine the concentration of cell-associated ions in centrifuged cell pellets. As reviewed in [30] and [31], those methods are error-prone and even small mistakes in the determination of the intracellular and extracellular water volumes result in large deviations in the respective ion concentrations. Another approach to measure intracellular concentrations of Na^+^, K^+^, and Cl^−^ is based on X-ray microanalysis, in which ion content and cell volume are microscopically estimated [32]. Both approaches could not be successfully applied to heterotrophic protists and generally led to cell rupture and distortion of cell shapes, which impeded calculations of the cell volume. More realistic estimates of intracellular ion concentrations were obtained for the green alga *Dunaliella*, due to the physical separation of cells from the extracellular medium by passing them through cation-exchange minicolumns [33,34] and an approach based on ^23^Na-nuclear magnetic resonance (NMR) spectroscopy using a dysprosium tripolyphosphate complex as a Na^+^-shift reagent to discriminate between intracellular and extracellular Na^+^ [35]. Those studies, however, were based on cell assemblages requiring reliable determinations of cell numbers.

A promising approach for the identification of compatible solutes is high-resolution NMR spectroscopy, which is routinely used in prokaryotic research to investigate the salt-out strategy, but was rarely applied to salt-related microeukaryotic research in this respect. One study using this technique focused on the effect of UV-induced stress on the metabolome of the moderate halophile ciliate *Fabrea salina*. Results suggested the accumulation of ectoine (Ect) and glycine betaine (GB) as osmoprotectans in a late reaction to UV radiation [36].

We here provide, to our knowledge, the first experimental study that investigates the salt adaptation strategy in the halophilic heterotroph ciliate *S*. *salinarum* [37]. Ciliates belong to the dominating protistan groups in hypersaline environments [38–41] and are therefore of high ecological relevance, e. g., as grazers exerting a top-down control on bacterial biomass. The heterotroph ciliate *S*. *salinarum* shows a wide geographic distribution and has a broad growth range between 1% and 21% salinity, with optimal growth at 9%.

Using *S*. *salinarum* as a model organism, the goals of our study were to establish experimental methods to (1) detect, identify, and quantify intracellular compatible solutes in salt-adapted heterotroph protists using proton (^1^H)-NMR spectroscopy and (2) determine relative intracellular ion concentrations in living single cells using ion imaging. Additionally, we tested and compared the activity of two enzymes of *S*. *salinarum* and *Escherichia coli* under high- and low-salt conditions. We found that *S*. *salinarum* uses the compatible solutes GB and Ect rather than accumulating inorganic ions to counterbalance osmotic stress. Intracellular concentrations of GB and Ect increased significantly with an increasing external salinity. Addition of exogenous GB, Ect, and choline (Ch; precursor of GB) stimulated the growth of *S*. *salinarum* notably, indicating that the organism is both able to accumulate the solutes from the external medium and also, apparently, to synthesize them. Enzyme activity assays support the salt-out strategy, as enzyme activity was reduced at high salt conditions, but not affected by GB at molar concentrations.

## Results

### Detection and identification of compatible solutes in *S*. *salinarum*

**Fig 1:**
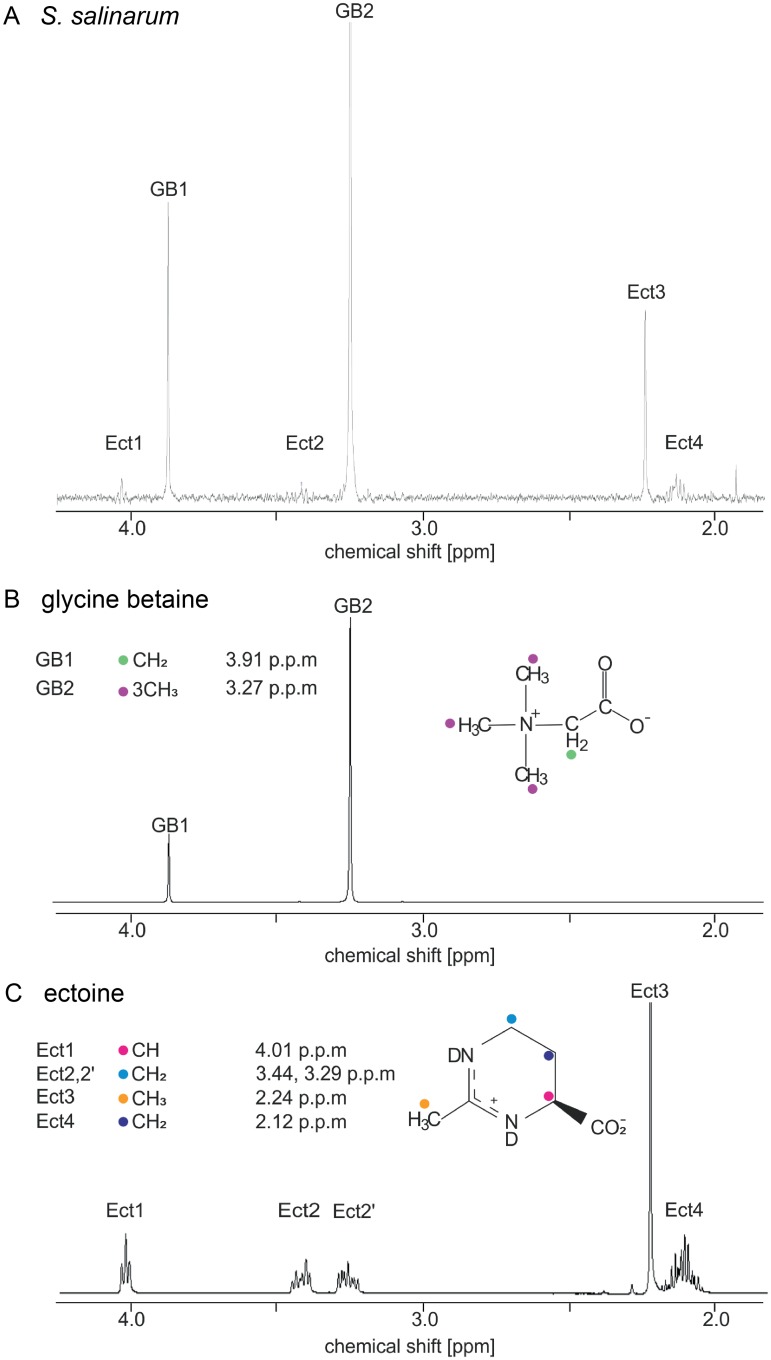
Identification of organic osmolytes using ^1^H-NMR spectroscopy. Comparison of ^1^H-NMR spectra of *S*. *salinarum* grown in ASW with 13% salinity (A), authentic GB (C_5_H_11_NO_2_) (B), and authentic Ect (C_6_H_10_N_2_O_2,_) (C). The chemical shifts of the peaks used for the assignment of GB were 3.91 ppm −CH_2_ and 3.27 ppm −(CH_3_)_3_, and for assigning Ect were 4.01 ppm −CH, 3.44 ppm and 3.29 ppm −CH_2_-ND, 2.44 ppm -CH_3_, and 2.12 ppm −CH_2_-CH. Peaks are color-coded in the spectra and in the chemical structures of GB and Ect. ASW, artificial seawater; C, carbon; D, deuterium; Ect, ectoine; GB, glycine betaine; ^1^H-NMR, proton nuclear magnetic resonance; H, hydrogen; N, nitrogen; O, oxygen; ppm, parts per million.

To investigate if *S*. *salinarum* implements the salt-out strategy, ^1^H-NMR spectroscopy was applied to detect and identify potential compatible solutes. Chemical shifts and coupling constants of all obtained ^1^H-NMR spectra from *S*. *salinarum* were compared to those obtained from authentic compatible solute spectra recorded under identical conditions, revealing the presence of GB and Ect (Fig 1). Separate addition of authentic GB and Ect to *S*. *salinarum* samples confirmed our results (S1 and S2 Figs). ^1^H-NMR spectra of ethanolic cell extracts gave NMR spectra as described above (S3 Fig), indicating that no cell material was lost during ^1^H-NMR sample preparations. To avoid spurious ethanol peaks, only nonethanolic extracts were analyzed.

Because our model organism *S*. *salinarum* is a bacterivore ciliate, it cannot be cultivated in axenic cultures. Our cultures, therefore, included a mixture of known indigenous bacteria (S1 Table) from the original sampling site serving as a food source. The main purpose of ^1^H-NMR controls was to exclude the possibility of detected compatible solutes originating from these bacteria. Our control experiments verified that the detected compatible solutes derive from *S*. *salinarum* cells and not from residual food bacteria. Neither osmolyte peaks could be detected in the bacterial ^1^H-NMR *s*pectra (S4A Fig) nor any food bacteria in the external medium (S4B Fig) or in the food vacuoles of starved *S*. *salinarum* cells (S4C Fig).

### Osmo-dependent accumulation of GB and Ect

**Fig 2:**
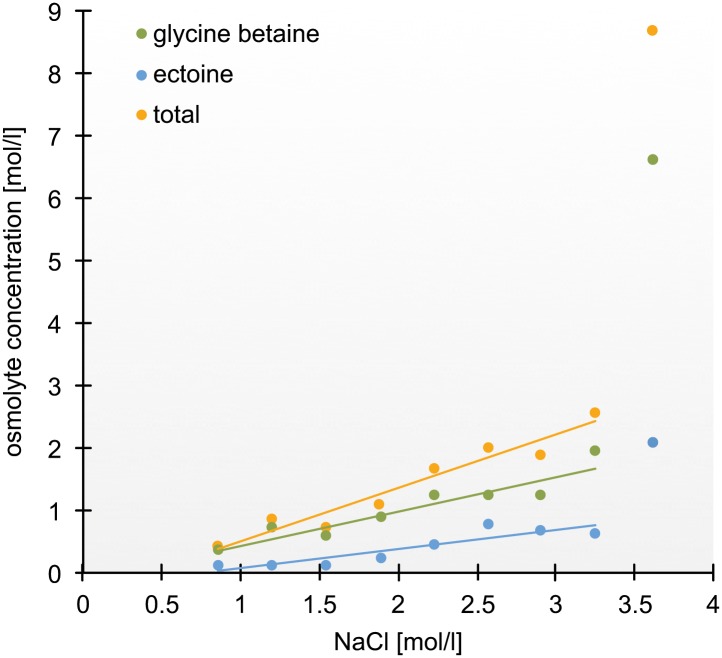
Relationship between intracellular concentrations of the organic osmolytes GB and Ect, as well as the sum of both (total) and increasing extracellular concentrations of NaCl, respectively. Both organic osmolytes, separately and in total, show a linear correlation with external NaCl concentrations up to 3.25 mol/l NaCl (GB: R^2^ = 0.85, *p* = 0.001; Ect: R^2^ = 0.78, *p* = 0.002; total: R^2^ = 0.92, *p* = 0.000). Raw data for this figure can be found in S1 Data. Ect, ectoine; GB, glycine betaine; NaCl, sodium chloride.

The compatible solutes GB and Ect were quantified by integrating the peak areas with respect to an internal standard (trimethylsilylpropanoic acid [TMSP]) along a salinity gradient (0.85 mol/l–3.59 mol/l NaCl). Average intracellular concentrations of GB (c(GB)) increased with increasing external salinity from 0.35 mol/l (± 0.2) at a salinity of 0.85 mol/l NaCl to up to 6.35 mol/l (± 1.0) at a salinity of 3.59 mol/l NaCl (Fig 2) (S5A Fig). Intracellular Ect concentration (c(Ect)) was as low as 0.09 mol/l (± 0.1) at an external NaCl concentration of 0.85 mol/l and increased to 2 mol/l (± 0.6) at a salinity of 3.59 mol/l NaCl (Fig 2) (S5B Fig). Between 3.25 mol/l NaCl and 3.59 mol/l NaCl, intracellular GB and Ect concentrations changed by a factor of three (otherwise: < 2). External NaCl concentration and average intracellular concentrations of GB and Ect, respectively, revealed a significant linear relationship (GB: R^2^ = 0.85, *p* = 0.001; Ect: R^2^ = 0.78, *p* = 0.002; Fig 2) between 0.85 mol/l NaCl and 3.25 mol/l NaCl. Total intracellular osmolyte concentration (sum of GB and Ect) and external NaCl concentration showed an even stronger linearity (R^2^ = 0.92, *p* = 0.000). Including the intracellular osmolyte concentrations measured at an external NaCl concentration of 3.59 mol/l lowered the coefficient of determination (GB: R^2^ = 0.46, *p* = 0.027; Ect: R^2^ = 0.64, *p* = 0.006; total: R^2^ = 0.51, *p* = 0.018).

As revealed by Welch’s *t* test (S5A Fig), significant changes in c(GB) occurred between 5% and 7% salinity (c(GB)_7%_ = 0.72 mol/l [± 0.3]; *p* = 0.032), between 11% and 13% salinity (c(GB)_11%_ = 0.85 mol/l 7 [± 0.3], c(GB)_13%_ = 1.23 mol/l [± 0.3]; *p* = 0.032), between 17% and 19% salinity (c(GB)_17%_ = 1.21 mol/l [± 0.5], c(GB)_19%_ = 1.94 mol/l [± 0.4]; *p* = 0.000), and between 19% and 21% salinity (c(GB)_21%_ = 6.35 mol/l [±1.04]; *p* = 0.000). Significant changes in c(Ect) along the salinity gradient appeared between 11% and 13% salinity (c(Ect)_11%_ = 0.23 mol/l [± 0.1], c(Ect)_13%_ = 0.45 mol/l (± 0.1); *p* = 0.007) and 19% and 21% salinity (c(Ect)_19%_ = 0.64 mol/l [± 0.2], (c(Ect)_21%_ = 2 mol/l (± 0.6); *p* = 0.002) (S5B Fig).

In total, c(GB) were at least two times higher than c(Ect) at all salinities (S6 Fig).

### Effect of exogenous osmolytes on growth efficiency of *S*. *salinarum*

**Fig 3:**
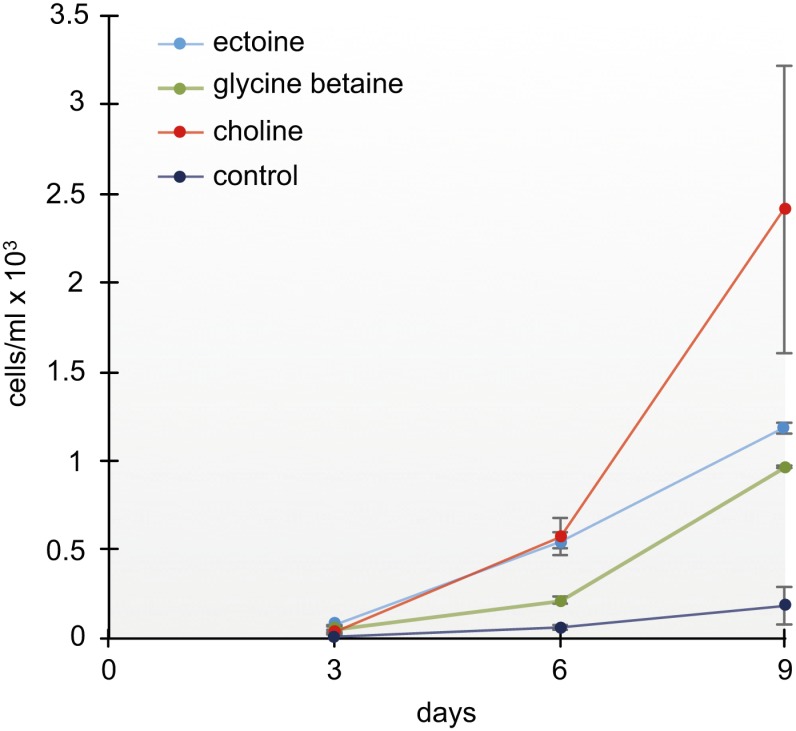
Effect of exogenously supplied compatible solutes on *S*. *salinarum* cell growth. Cultures treated with Ect, GB, and its precursor Ch (final concentration: 1 mM each) show an increased growth compared to the control cultures. Cell numbers at the beginning of the experiment were 500 cells ml^−1^. Raw data for this figure are deposited in S1 Data. Ch, choline; Ect, ectoine; GB, glycine betaine.

To examine whether *S*. *salinarum* is able to accumulate compatible solutes from the external medium, cultures were incubated with GB, Ect, and Ch, whereas Ch acts as a precursor in the synthesis pathway of GB. After nine-d growth, ciliate cell abundance in the GB-enriched culture medium was increased compared to the control cultures (Fig 3). Control cultures included on average 280 cells ml^−1^ (± 181), whereas the GB-enriched culture included 983 cells ml^−1^ (± 32). The increase of cell abundance was pronounced but not significant (*p* = 0.85). In the Ch-enriched cultures cell abundances (3,247 cells ml^−1^ ± 1557) were significantly increased compared to the control (*p* = 0.004) and compared to the GB-enriched cultures (*p* = 0.027). Exogenously provided Ect increased the cell density up to 1,380 cells ml^−1^ (± 28). The increase of cell abundances was pronounced but not significant compared to the control and the GB treatment (*p* > 0.05).

### Oxidation of 1,2-^13^C_2_-Ch to 1,2-^13^C_2_-GB by *S*. *salinarum*

**Fig 4:**
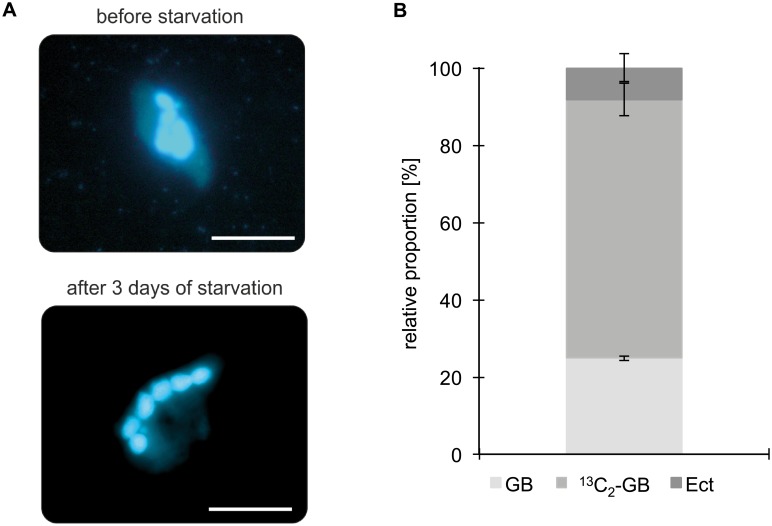
(A), DAPI-staining of *S*. *salinarum* before (top) and after (bottom) three d of starvation. Before starvation, numerous bacteria can be observed in the external medium and the cytoplasm of *S*. *salinarum*. The six macronuclei of *S*. *salinarum* can clearly be identified in both pictures. Bars correspond to 50 μm. (B), Ratio of GB:1,2-^13^C_2_-GB:Ect concentrations in starved *S*. *salinarum* cells after a three-d incubation with 1,2-^13^C_2_-Ch in 13% salinity (*n* = 3). Raw data for the bar chart can be found in S1 Data. C, carbon; Ch, choline; Ect, ectoine; GB, glycine betaine.

The dominant pathway to synthesize GB is by a two-step oxidation of Ch via betaine aldehyde. This oxidative pathway can occur with different enzymes, e.g., in plants, Ch is oxidized to betaine aldehyde by a ferredoxin-dependent Ch monooxygenase [42] and betaine aldehyde is mediated by a soluble, nicotinamide adenine dinucleotide (NAD^+^)-linked betaine aldehyde dehydrogenase into GB [43]. However, the enhanced growth of *S*. *salinarum* with exogenously supplied Ch indicated a preference for the GB-precursor Ch over GB itself (see growth experiments above). Because this would point to a potential GB synthesis based on the conversion of Ch to GB, we conducted an uptake experiment with labelled ^13^C_2_-Ch. In a first step, *S*. *salinarum* cultures were starved to exclude the possibility of bacterial contaminations. After three d of starvation, DAPI staining revealed bacteria-free cultures (Fig 4A). ^1^H-NMR spectroscopy was conducted to examine if *S*. *salinarum* is able to take up and oxidize exogenously provided ^13^C_2_-Ch into ^13^C_2_-GB. ^1^H-NMR spectroscopy revealed that the 1,2-^13^C_2_-labeled Ch was internalized and converted to 1,2-^13^C_2_-GB in all replicate cultures (S7 Fig). The ratio of GB:1,2-^13^C_2_-GB:Ect showed no significant differences between the individual replicates and was on average 3:8:1 (Fig 4B).

### Relative intracellular Na^+^ and K^+^ concentration

To study the intracellular Na^+^ and K^+^ concentrations of *S*. *salinarum*, we established a microscopy-based approach, which relies on ion-specific fluorescent dyes (ion imaging). For the relative fluorescence measures of the cytoplasm, only the cytoplasm was integrated as a region of interest, not the vacuole-like structures. Results are given as normalized corrected relative fluorescence intensities (CRF).

**Fig 5:**
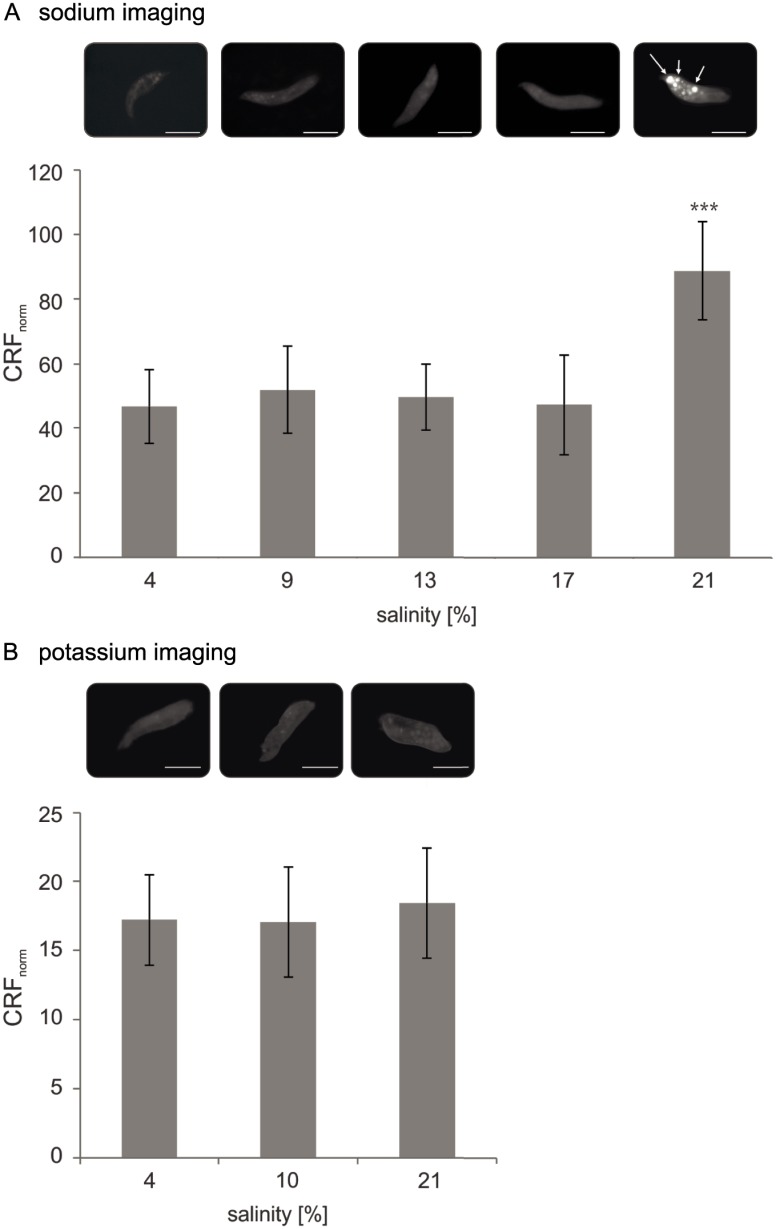
Relative intracellular ion concentrations in *S*. *salinarum* at increasing external salinity. (A), Na^+^ imaging revealed a significantly increased accumulation of Na^+^ in the cytoplasm of *S*. *salinarum* at 21% salinity. Additionally, Na^+^ entrapments were observed in vacuole-like structures at this salt concentration (indicated by white arrows). (B), K^+^ imaging revealed steady K^+^ concentrations in the cytoplasm of *S*. *salinarum*. Relative fluorescence intensity was determined for the cytoplasm only (*n* = 10). Raw data are deposited in S1 Data. Generic pictures are shown for each treatment and salinity, respectively. Bars correspond to 50 μm. CRF, corrected relative fluorescence intensity; CRF_norm_, normalized CRF; K, potassium; Na, sodium.

Na^+^ imaging of *S*. *salinarum* cells grown in the respective salinities (long-time adapted cells) revealed stable Na^+^ concentrations in the cytoplasm of *S*. *salinarum* at external salinities between 3.8% and 17% (CRF_4%_ = 46.70 ± 12; CRF_9%_ = 52.03 ± 14, CRF_13%_ = 49.70 ± 10, CRF_17%_ = 47.30 ± 15), and a significant Na^+^ increase at 21% salinity (CRF_21%_ = 88.70 ± 15; Fig 5A). Additionally, vacuole-like structures containing elevated Na^+^ concentrations were observed in the *S*. *salinarum* cells exposed to 21% salinity (Fig 5A). K^+^ imaging of long-time adapted *S*. *salinarum* cells revealed steady cytoplasmic K^+^ concentrations with increasing external salinity (CRF_4%_ = 17.17 ± 3; CRF_10%_ = 17.04 ± 4; CRF_21%_ = 18.40 ± 4; *p* > 0.05) (Fig 5B).

To test whether the observed increase in Na^+^ concentration with increasing salinity in long-time adapted cells was a result of passive ion influx or rather a product of active regulated uptake (i.e., presence of specific membrane transporters), short-time adaptation experiments were conducted with single *S*. *salinarum* cells exposed to increasing salt concentrations. Na^+^ imaging with short-time adapted cells showed no significant changes in Na^+^ concentrations in the cytoplasm. The relative fluorescence intensity was on average 12.48 ± 2. After a salinity increase up to 18%, the cells busted due to the high osmotic pressure (S8 Fig). Increasing the adaptation time to 10 min to allow for a longer equilibration resulted in slightly lower average relative fluorescence intensity (8.62 ± 1; S8B Fig), but did also not show any significant increase in Na^+^ concentration.

### Effect of salinity on enzyme activity

High intracellular Na^+^ concentrations can inhibit enzymes if the proteomes of the cells are not sufficiently adapted. In a third experimental approach, we investigated the kinetic performance of two cytosolic enzymes under different salt and osmolyte conditions and compared their protein charge with respective proteins of *E*. *coli* to get a hint of whether *S*. *salinarum* might possess an acidic proteome.

The first enzyme, malate dehydrogenase (MDH) catalyses the interconversion of L-malate to oxaloacetate using the reduction of NAD^+^ to NADH (nicotinamide adenine dinucleotide). Whereas prokaryotes contain only a single MDH, eukaryotes have several MDHs, including a cytoplasmic form. Cytosolic MDH assists the malate-aspartate shuttle so that reducing equivalents can pass through the mitochondrial or peroxisomal membrane for cellular processes. Our phylogenetic analysis (using the dataset from [44]) shows that MDH from *S*. *salinarum* and the highly similar MDHs from the ciliates *Stylonychia lemnae* (79% identity) and *Oxytricha trifallax* (77% identity) belong to the clade I subset of cytosolic and plastidial MDHs, including the human cytosolic MDH (S9 Fig). MDH from *S*. *salinarum* only shows a weak sequence similarity with the human mitochondrial MDH (23% identity) and *E*. *coli* MDH (21% identity). Inspection of the DNA sequence 3ʹ of the stop codon of the *S*. *salinarum* MDH gene did not reveal evidence for a read-through stop codon followed by a peroxisomal serine-lysine-leucine targeting sequence [45]. Nevertheless, MDH enzymes have similar kinetic properties and share a common catalytic mechanism, which is reflected by a high degree of structural similarity [46] between the various MDH clades. *S*. *salinarum* MDH has all highly conserved amino acid residues involved in substrate binding (R116, R122, R186, N131) of the active site (H187) and for NAD^+^ binding site (N129), which leaves no doubt about the assignment as genuine MDH enzyme.

The second enzyme investigated, isocitrate dehydrogenase (ICDH), catalyses the oxidative decarboxylation of isocitrate to 2-oxoglutarate and requires NAD^+^ or nicotinamide adenine dinucleotide phosphate (NADP^+^), producing NADH and NADPH, respectively. Whereas *E*. *coli* has only one form, humans contain three classes of ICDH isoenzymes: mitochondrial NAD^+^-dependent, mitochondrial NADP^+^-dependent, and cytosolic NADP^+^-dependent ICDH. ICDH from *S*. *salinarum* and the ciliates *S*. *lemnae* and *O*. *trifallax* ICDHs (both 78% identity) are phylogenetically related to homodimeric NADP^+^-dependent ICDHs [47] such as the human cytoplasmic enzyme (68% identity, S10 Fig), which has a significant role in NADPH production. The cytosolic form from many eukaryotes can also be localized to the peroxisomes through a C-terminal peroxisomal targeting sequence. Analysis of the *S*. *salinarum* DNA sequence neither identified C-terminal and N-terminal peroxisome-targeting sequences for peroxisomal import, nor a clear mitochondrial targeting sequence, supporting cytosolic localization.

**Fig 6:**
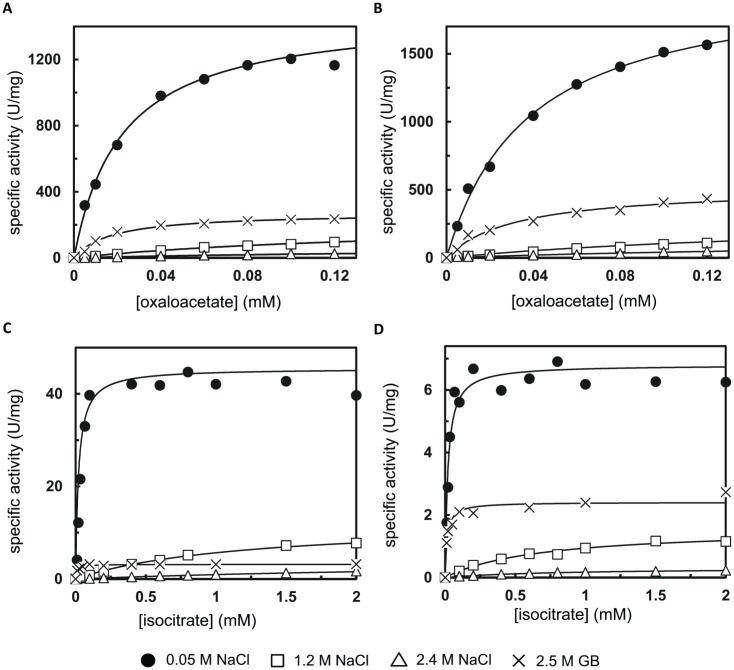
Enzyme activities of *E*. *coli* and *S*. *salinarum* MDHs and ICDHs. Enzymatic activity of *E*. *coli (*A, C) and *S*. *salinarum* (B, D) as a function of oxaloacetate or isocitrate concentration, respectively, in the presence of NaCl or GB. For all conditions, the buffer contained 32.5 mM Tris/HCl, pH 7.5. Nonlinear least-squared fits to the Michaelis-Menten equation are shown. See Table 1 for values of K_m_, V_max_, and the specificity constant, and S1 Data for underlying raw data. GB, glycine betaine; HCl, hydrogen chloride; ICDH, isocitrate dehydrogenase; MDH, malate dehydrogenase; NaCl, sodium chloride; Tris, tris(hydroxymethyl)aminomethane.

ICDH and MDHs were overexpressed in *E*. *coli* cells and purified using nickel-nitrilotriacetic acid (Ni-NTA) chromatography. Most of these proteins appeared in the soluble fraction upon fractionation. After sodium dodecyl sulfatepolyacrylamide gel electrophoresis (SDS-PAGE), clear bands corresponding to calculated molecular masses (MDH_*E*.*coli*_: 34.2 kDa (kilodalton), MDH_*S*.*salinarum*_: 40.7 kDa, ICDH_*E*.*coli*_: 48.2 kDa, ICDH_*S*.*salinarum*_: 48.7 kDa) were observed in the purified proteins (estimated purity 95%–98%, S11 Fig). The effect of NaCl at low (0.05 M) and high salt conditions (1.2 and 2.4 M NaCl) on the enzymatic activity was analysed by determination of the Michaelis Menten constants (Table 1). MDH_*E*.*coli*_ at 0.05 M NaCl presented a hyperbolic curve, with specific activity (V_max_ of 1,486 U mg^−1^) and K_*m*_ value for oxaloacetate (0.022 mM), values typical for this class of enzyme [48,49] (Fig 6A and Table 1). At a low salt concentration, MDH_*S*.*salinarum*_ presents an even higher specific activity (V_max_ = 2,062 U mg^−1^) than the *E*. *coli* enzyme, with a comparable K_m_ value for oxaloacetate (0.038 mM) to the microbial enzyme (Fig 6B). When the salt concentration in the buffer was elevated to 1.2 M, activity of the MDH enzymes from both organisms strongly decreased and the K_*m*_ values increased almost tenfold (Table 1). At 2.4 M NaCl, only a residual MDH activity of 5% and 7% for the *E*. *coli* and *S*. *salinarum* enzyme, respectively, remained. It is known that *E*. *coli* can accumulate K^+^ up to 0.9 M in the initial phase of osmoadaptation, followed by intracellular accumulation of compatible solutes, e.g., GB [50]. Our results show that addition of GB at high concentration (2.5 M) to the buffer impaired MDH_*E*.*coli*_ activity (18% residual activity) but to a much lower extent than with 2.4 M NaCl. Under the same condition, MDH_*S*.*salinarum*_ retained more activity (25%). GB had no remarkable effect on the K_*m*_ values.

**Table 1:** Summary of enzyme kinetic of MDH and ICDH from *E*. *coli* and *S*. *salinarum*.

	Salt or Osmolyte (M)	K_m_ (mM)	V_max_ (U mg^−1^)	Specifity Constant (U mg^−1^ mM^−1^)	Osmolyte Performance (V_max_/K_*m*_ relative to 0.05 M NaCl)
MDH_*E*.*coli*_	0.05 M NaCl	0.022	1,486	67,443	100
	1.2 M NaCl	0.163	227	1,393	2.1
	2.4 M NaCl	0.26	78	306	0.5
	2.5 M GB	0.018	273	15,478	23
MDH_*S*.*salinarum*_	0.05 M NaCl	0.038	2,062	54,832	100
	1.2 M NaCl	0.20	314	1,568	3
	2.4 M NaCl	0.25	144	578	1.1
	2.5 M GB	0.031	519	16,527	30
ICDH_*E*.*coli*_	0.05 M NaCl	0.021	46	2,167	100
	1.2 M NaCl	1.2	12.3	10	0.5
	2.4 M NaCl	3.9	4.7	1.2	0.1
	2.5 M GB	0.009	3.2	369	17
ICDH_*S*.*salinarum*_	0.05 M NaCl	0.019	6.8	353	100
	1.2 M NaCl	0.68	1.6	2.3	0.7
	2.4 M NaCl	0.82	0.32	0.4	0.1
	2.5 M GB	0.014	2.4	170	48

GB, glycine betaine; ICDH, isocitrate dehydrogenase; MDH, malate dehydrogenase; NaCl, sodium chloride.

A qualitatively similar picture was observed for ICDH (Fig 6C and 6D and Table 1). At low salt conditions, ICDH_*E*.*coli*_ had a V_*max*_ of 46 U mg^**−**1^ and a K_m_ for DL-isocitrate of 0.021 mM, values comparable to those found for many ICDHs [51]. Interestingly, ICDH_*S*.*salinarum*_ had a similar K_m_ (0.010 mM) but was less active (V_max_ of 6.8 U mg^**−**1^), like some eukaryotic ICDHs [52]. It is possible that an allosteric activation mechanism regulates the *S*. *salinarum* enzyme. As observed for all ICDHs, ICDH_*S*.*salinarum*_ also required magnesium (Mg^2+^) ions to be active. ICDH from *E*. *coli* and from *S*. *salinarum* were much less active upon increasing the salt concentration. At 1.2 M NaCl, 27% and 23% of the V_max_ was observed for *E*. *coli* and *S*. *salinarum* enzymes, whereas at 2.4 M NaCl residual activities of 10% and 5% were found. ICDH_*S*.*salinarum*_ had a better performance in 2.5 M GB than ICDH_*E*.*coli*_, as the enzyme retained about 35% of its activity in comparison with 7% for the *E*. *coli* protein. Again, GB had no remarkable effect on the K_*m*_ values (Table 1). These findings contrast with the kinetic properties of ICDHs purified from the halophiles *Haloferax volcanii* and *Halobacterium salinarum*, which are salt-activated enzymes reaching a V_*max*_ comparable to the *E*. *coli* enzyme [53,54].

Taken together, the in vitro activity measurements support the salt-out strategy as indicated by the ^1^H-NMR experiments of *S*. *salinarum*.

## Discussion

### GB and Ect are major compatible solutes in *S*. *salinarum*

Using ^1^H-NMR spectroscopy, we were able to provide the first experimental detection of compatible solutes and their accumulation with increasing salinity in heterotrophic protists. Based on our results, we can conclude that *S*. *salinarum* utilizes two major compatible solutes, GB and Ect (Fig 1), to combat high salt concentrations in its environment. Furthermore, our approach revealed an accumulation of both compatible solutes with increasing salinity. Thus, the concentration of both seems to be regulated in dependence of different salt concentrations (Fig 2) (S5 Fig).

**Fig 7:**
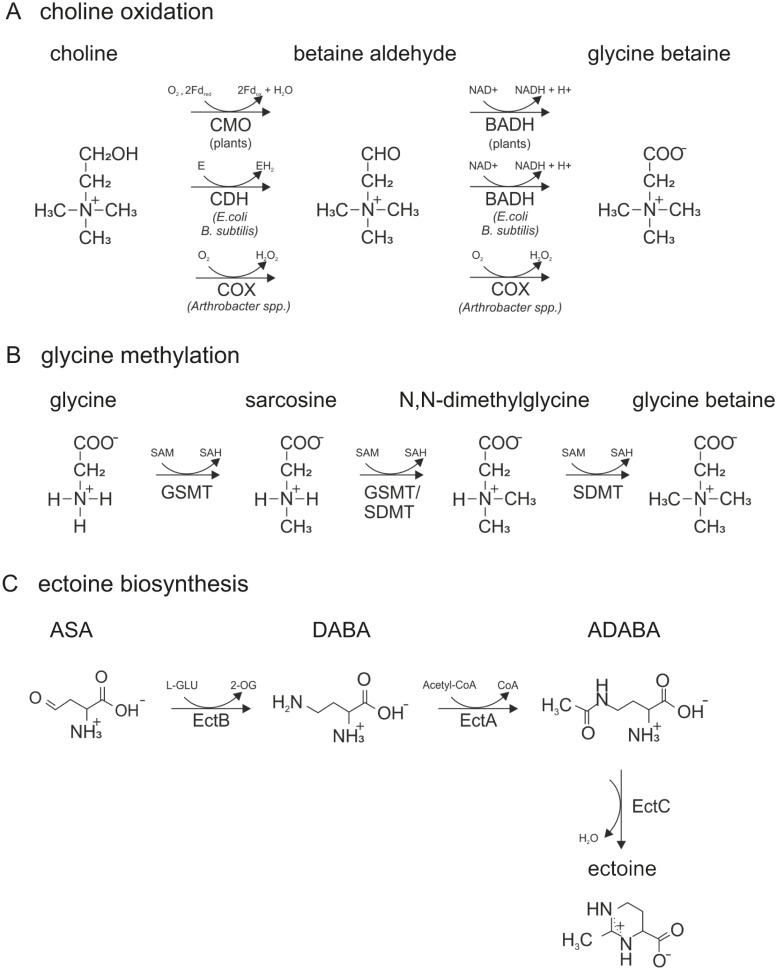
GB and Ect synthesis pathways. (A), Ch oxidation. Depending on the organism, the two-step oxidation is catalysed by different enzymes. In plants, Ch is oxidized by CMO to betaine aldehyde, which is subsequently oxidized by BADH. In some marine invertebrates and bacteria, Ch is oxidized by CDH to betaine aldehyde, which is then oxidized by BADH to GB. Some *Arthrobacter* spp. are able to oxidize both steps with COX. (B), Glycine methylation. Some extremely halophilic bacteria produce GB by a three-step methylation of glycine. GSMT and SDMT transfer the methyl group of SAM to two different types of amines, sarcosine and *N*,*N*-dimethylglycine. (C), Ect biosynthesis in *Halomonas elongata*. ASA is converted into L-2,4-DABA, which is then acetylated to form Nγ-acetyl-L-2,4-ADABA. The last step is the cyclization of ADABA to Ect. The genes coding for the biosynthesis are the three genes EctABC [63]: EctA codes for diaminobutyric acid acetyltransferase, EctB codes for the diaminobutyric acid aminotransferase, and EctC codes for Ect synthase. 2-OG, 2 oxoglutarate; ADABA, diaminobutyrate; ASA, aspartate semialdehyde; BADH, betaine aldehyde dehydrogenase; C, carbon; CDH, choline dehydrogenase; Ch, choline; COX, Ch oxidase; CMO, choline monooxygenase; DABA, diaminobutyric acid; Ect, ectoine; GB, glycine betaine; H, hydrogen; GSMT, glycine sarcosine methyltransferase; L-Glu, L-glutamate; O, oxygen; N, nitrogen; SAH, S-adenosylhomocysteine; SAM, S-adenosylmethionine; SDMT, sarcosine dimethylglycine methyltransferase.

The dominant compatible solute throughout all tested salinities was GB (*N*,*N*,*N*-trimethylglycine), which is one of the most common osmoprotectants in halophilic bacteria and archaea of diverse phylogenetic affiliation [55], halotolerant plants, as well as algae [56,57], and was also detected in halotolerant heterokont thraustochytrids [58]. Usually, organisms accumulate GB actively from culture media because many of the regular components, like yeast extract, contain this organic solute [57]. Nevertheless, there are two generally different pathways describing how GB can be synthesized (Fig 7A and 7B). In almost all cases, it is synthesized by a two-step oxidation of Ch via betaine aldehyde. This oxidative pathway can occur with different enzymes. In plants, Ch is oxidized to betaine aldehyde by a ferredoxin-dependent Ch monooxygenase [42] and betaine aldehyde is mediated by a soluble, NAD^+^-linked betaine aldehyde dehydrogenase into GB [43]. Both enzymes are located in plant chloroplasts [59]. In bacteria, including *E*. *coli*, a membrane-bound electron transfer-linked Ch dehydrogenase oxidizes Ch to betaine aldehyde, which is subsequently oxidized to GB by betaine aldehyde dehydrogenase [60]. Alternatively, some *Arthrobacter* spp. have a soluble Ch oxidase (COX) that carries out both oxidations [61]. In a completely different pathway, GB is synthesized via consecutive methylation of glycine to GB. However, as far as is known, this pathway is utilized by a few extremely halophilic bacteria only ([62], and references therein).

The second identified compatible solute Ect (1,4,5,6-tetrahydro-2-methyl-4-pyrimidinecarboxylic acid) is an almost equally wide distributed compatible solute throughout different halophilic and halotolerant bacteria [64–71]. Among the so far investigated compatible solutes, Ect displays the most powerful stabilizing properties in a variety of different enzymatic processes [72]. It features distinct protective effects against the stress factors heat, cold, and high salt concentrations in diverse prokaryotes [72–74] and is known to be accumulated by the moderate halophilic ciliate *F*. *salina* to protect the organism against UV radiation [36]. Ect can be both, accumulated from the environment or synthesized de novo [75]. The biosynthesis of Ect has been intensively studied in the bacterium *Halomonas elongata* [64] (Fig 7C). In a first step, aspartate-β-semialdehyde is converted to L-2,4-diaminobutyric acid catalyzed by diaminobutyric acid aminotransferase. Subsequently, L-2,4-diaminobutyric acid is converted by diaminobutyric acid acetyltransferase into Nγ-acetyl-L-2,4-diaminobutyrate. Ultimately, N-acetyl-L-2,4-diaminobutyrate is converted into Ect via Ect synthase [75]. For a longer period of time, Ect synthesis was thought to be restricted to bacteria and a few archaea [76], but only recently expression patterns of genes potentially involved in the synthesis of Ect and 5-hydroxyectoine (a derivative of Ect) were recognized in the predicted proteomes of the two halophilic flagellates *H*. *seosinensis* and *P*. *kirbyi* [25].

The accumulation of different solutes, instead of only one, is known from most halotolerant and halophilic prokaryotes (e.g., [77,78]). Occasionally it is a combination of zwitterions—like GB and Ect—but more frequently of several solutes with the same net charge (as reviewed in [78]). At first glance, our results suggest the exclusive accumulation of GB and Ect, which has been observed in a few halophilic and halotolerant prokaryotes only [66,79,80]. If this would be true, we would expect that the sum of the osmolyte concentrations is osmotically equivalent to the external NaCl concentration. For *S*. *salinarum*, however, we measured an internal osmolyte concentration of ca. 2 M at an external NaCl concentration of 3 M (Fig 2). This disparity might be explained by the following reason: calculations of the cell volumes of *S*. *salinarum* were based on size measurements of individual cells from examined organisms using scanning electron microscopy (SEM) pictures. The estimation of an exact cell volume, however, is heavily dependent on the applied mathematics, as discussed previously by Sun and Liu [81]. Even the most adequate model cannot completely mirror the exact cell shape and profile and thus one should be aware that calculations based on cell width, length, and height are only approximations. Additionally, those mathematic models do not consider “dead spaces” occupied by e.g., oil bodies and/or starch granules, which can be detected in *S*. *salinarum* cells [37]. Those structures can account for a notable proportion of the total cell volume. We therefore attribute the difference in estimated internal and known external osmolality to the uncertainty in the calculation of the cell volume.

Our growth experiments with exogenously provided GB and Ect showed an increase of *S*. *salinarum* densities (Fig 3). Interestingly, the addition of the GB precursor Ch resulted in a 12 times higher cell number when compared to the control culture (without any exogenous solutes). This points to a possible GB synthesis by oxidation of Ch to GB. It should be noted that the growth experiments were influenced by the number of food bacteria in the cultures, which could have benefited from exogenously provided solutes. Therefore, a direct approach was conducted by adding labeled 1,2-^13^C_2_-Ch to the medium of starved, bacteria-free *S*. *salinarum* cultures (Fig 4). Using ^1^H-NMR spectroscopy, indeed, 1,2-^13^C_2_-GB was detected, probably resulting from the oxidation of 1,2-^13^C_2_-Ch to 1,2-^13^C_2_-GB (Fig 4) (S7 Fig). Our results demonstrate for the first time that Ch oxidation might well occur in heterotrophic protozoa. Analysis of the *S*. *salinarum* transcriptome could shed light on the enzymes involved in the conversion. Although the Ect biosynthesis was not experimentally investigated in this study, we observed an increased Ect concentration and therefore importance at higher salinities (S6 Fig). This is supported by the recent transcriptome data on two halophilic flagellates, emphasizing a de novo synthesis of Ect in halophilic protists [25].

### *S*. *salinarum* does not intracellularly accumulate Na^+^ and K^+^ ions

Although even low concentrations of Na^+^ are toxic for most living cells [82], a few halophilic microorganisms, in particular some *Halobacteria* [83] and the yeast *D*. *hansenii* [84], require high intracellular concentrations of Na^+^ for normal enzymatic activities. For *S*. *salinarum*, ion imaging revealed no significant differences in the relative intracellular cytoplasmatic Na^+^ concentration (Fig 5A) at lower salinities (4%–17%). However, Baxter and Gibbons [85] pointed out that a constant low intracellular ionic environment can only be achieved by energy-dependent mechanisms. As high extracellular NaCl concentrations lead to a constant influx of Na^+^ ions because of a not completely impermeable membrane and the additional influx of Na^+^ during cotransport of certain substrates or amino acids [86], it is to assume that *S*. *salinarum* requires some Na^+^ extrusion mechanisms in its cell membrane. So far, there are two major mechanisms for several halotolerant and halophilic prokaryotes (e.g., [86–88]), plants (e.g., [89–91]), and halotolerant yeasts (e.g., [21,82,92–94]) described: the activity of Na^+^/H^+^ antiporter and the presence of a primary respiration-driven Na^+^ pump. Short-time ion imaging experiments revealed that *S*. *salinarum* was not able to adapt to salinities higher than 16% in a time span of 5 to 10 min. It might be possible, that the observed Na^+^ exclusion is not achieved by increased activity of certain transport mechanisms, but rather by an increased number of transporters, which requires time-consuming de novo synthesis. An overexpression of genes encoding for a Na^+^ extrusion pump triggered by high NaCl concentrations was, for instance, found in the salt-loving yeast *D*. *hansenii* [21]. However, this hypothesis should be proofed, e.g., by a comparative transcriptome analysis of *S*. *salinarum* at low and high external salt concentration.

*S*. *salinarum*, shifted to higher salinities (21%), showed a significant increase in Na^+^ concentration in the cytoplasm (Fig 5A). Because *S*. *salinarum* does not tolerate external salinities higher than 21%–23%, this increase of intracellular Na^+^ may be explained by the failure to actively exclude the high concentration of salt ions. Also, the strong increase of osmolytes (Fig 2) (S5 Fig) at this salinity may be a result of a penultimate attempt to counterbalance the elevated influx of salt ions. Instead, *S*. *salinarum* seems to accumulate the excess of Na^+^ in vacuole-like structures (Fig 5A, indicated by white arrows) to maintain a chemically balanced cytoplasm. These structures may be a mechanism to sequestrate intracellular Na^+^. As a fact, the Na^+^/H^+^ exchanger (*NHX1*), which is known from yeast [95] and higher plants [96], transports Na^+^ into vacuolar and/or prevacuolar compartments. In plants, the vacuolar sequestration of Na^+^ not only lowers Na^+^ concentrations in the cytoplasm but also contributes to osmotic adjustment to maintain water uptake from saline solutions [97]. Besides vacuoles, even plastids and mitochondria accumulate Na^+^ and thereby additionally contribute to the overall subcellular compartmentalization of Na^+^. Furthermore, the overexpression of specific Na^+^/H^+^ transporters localized, e.g., in tonoplast membranes and the increased expression of vacuolar H^+^/ATPase components due to osmotic stress were reported in various plants and are known to substantially enhance plant salt tolerance [96,98–102].

K^+^ concentrations did not change with increasing salinities, neither in the cytoplasm nor in the vacuoles (Fig 5B). This, together with steady intracellular Na^+^ concentrations between 4%–17% external salinity, perfectly fits the assumption that *S*. *salinarum* is a salt-out strategist (using compatible solutes) to balance its osmotic potential. Salt-out strategists need to maintain a stable high intracellular K^+^/Na^+^ ratio for several physiological functions like osmotic regulation, protein synthesis, enzyme activation or the maintenance of the plasma membrane potential [103]. Unfortunately, the ion imaging approach only informs about relative internal ion concentration ratios between different external salinities. A focused analysis of ion homeostasis in the transcriptome of *S*. *salinarum* will provide more information.

### *S*. *salinarum* protein activity is reduced at high salt concentrations

Species implementing the salt-in strategy are frequently characterized by an excess of acidic amino acids such as glutamate and aspartate, a low content of alkaline amino acids such as lysine and arginine, and a low content of hydrophobic amino acid residues [104]. Acting in concert, these proteome modifications help to prevent aggregation and to stabilize proteins, and also to maintain a hydration shell and the solubility of proteins [14,105,106]. Our results indicate that this peculiarity is not shared by the here investigated ciliate MDH and ICDH proteins. As judged by the calculated isoelectric point (pI) values (MDH = 6.08; ICDH = 8.49), the proteins from *S*. *salinarum* are neutral or slightly positively charged at physiological pH. Halotolerant eukaryotes, e.g., *Tamarix tetragyna*, have MDH with decreased activity at increasing NaCl concentration [107]. As *S*. *salinarum*, these organisms use organic compatible solutes that allow flexibility with respect to the range of salt concentrations tolerated and do not require a high degree of adaptation of the intracellular enzymes. Biochemically, these enzymes do not greatly differ from those of nonhalophilic prokaryotes [108]. Taken together, our data clearly show that *S*. *salinarum* MDH and ICDH do not require high salt concentrations for their activity; contrarily, they are much less active at a NaCl concentration corresponding to optimal growth salinity. Compatible with our findings of molar concentrations of the compatible solute GB in *S*. *salinarum* cells, substrate affinity and activity of MDH and ICDH are unaffected or only mildly affected by 2.5 M GB.

This is, to our knowledge, the first experimental study on haloadaptation strategies of a halophilic heterotrophic protist, for which three approaches were established to investigate haloadaptation strategies, previously known only from archaea and bacteria. Our results suggest that the heterotroph ciliate *S*. *salinarum* is a salt-out strategist. This is based on the direct measurement of two compatible solutes, namely GB and Ect, the active exclusion of Na^+^ and K^+^ ions out of the internal cell environment and a reduced protein activity of two key enzymes at high salt conditions in *S*. *salinarum*. This study is pioneering the route towards a better understanding of haloadaptations in heterotrophic protists and lays the foundation for future research and scientific studies in this field.

## Materials and methods

### Cultivation of *S*. *salinarum*

The halophile ciliate *S*. *salinarum* [37] was isolated from a solar saltern pond in Ses Salines, Mallorca, Spain (Salinas d`Es Trenc, N 39° 21’ 16.11” E 3° 0’ 42.16”) with a salinity of 12%. Single ciliate cells were handpicked in a 5 μl volume and transferred into 2 ml of sterilized salt medium (artificial seawater, ASW; Instant Ocean, Aquarium Systems, Cleveland, Ohio; for chemical composition see [109]) to establish initial cell cultures. This way, a proportion of indigenous bacteria, required by the ciliates as food source, were also transferred into the initial culture. When ciliate cell density reached about 100 cells/ml, the initial culture was diluted with sterile salt medium to a volume of 15 ml. Subsequent cultures were established by dividing the initial culture, therefore always transferring *S*. *salinarum* cells as well as indigenous food bacteria into the new cultures. Ciliate cells were cultivated in different salt concentrations ranging from 3.8%–21% at room temperature (RT) with a 12 h light-dark cycle. All cultures were amended with one sterile wheat grain per 15 ml culture to support growth of indigenous bacteria. Further information about the indigenous food bacteria is supplied in S1 Table.

### ^1^H-NMR spectroscopy

^1^H-NMR spectroscopy was applied to detect and identify possible compatible solutes in *S*. *salinarum*. For this, cells were concentrated by centrifugation in a pear-shaped ASTM centrifuge tube (Lenz Laborglas GmbH & Co. KG, Wertheim, Germany) for 5 min at 400 g (Roto Silenta III, Hettich, Germany), followed by removal of the supernatant. Cells were checked for positive vitality under a stereo microscope (SZ-PT, Olympus, Tokyo, Japan) and then starved in sterile ASW medium for three d to reduce the total number of food bacteria. After starvation, ciliate abundance was determined by cell counting and samples were filtered immediately onto Isopore membranes (25 mm; 5 μm; Millipore, Schwalbach am Taunus, Germany). Cells were broken by shear forces in a Concentrator 5301 (Eppendorf AG, Hamburg, Germany) at maximum speed at 60 °C for 4 h. The samples were resuspended in 500 μl D_2_O, vortexed, and centrifuged for 20 min at 13.000 rpm (Hermle Z 233 MK, Hermle-Labortechnik, Wehingen, Germany). The supernatant was transferred into a new tube, the filter membrane was suspended with 400 μl D_2_O, and centrifuged again for 10 min at 13.000 rpm. Supernatants were mixed and centrifuged an additional time for 10 min. Finally, 600 μl sample was transferred to an NMR tube and mixed with TMSP and phosphate buffer (final concentration 6 mM K_2_HPO_4_; 4 mM KH_2_PO_4_). TMSP was added at a concentration of 500 mM as an internal chemical shift reference and for the quantification of organic osmolytes [110]. Measurements were done for six culture replicates growing in 5, 7, 9, 11, 13, 15, 17, 19, and 21% salinity.

To avoid losing any cellular substances by the extraction protocol described above, an ethanol extraction following the protocol of [64] but with minor modifications was also performed. Briefly, after filtration of cultures, cells were washed with ASW, resuspended in 20 ml of 80% (v/v) ethanol, vortexed for 5 min at RT, incubated for 2 h to extract the intracellular solutes and centrifuged again for 10 min at 13.000 rpm. The supernatant was retained for determination of compatible solutes. Cells were re-extracted as described above with 10 ml 80% ethanol. Cell extracts were combined and evaporated to dryness at 60 °C. Sample preparation for ^1^H-NMR spectroscopy followed the protocol described above.

### Identification and quantification of organic osmolytes

One-dimensional ^1^H-NMR spectra were recorded on a 400-MHz Bruker Advance III spectrometer (Bruker BioSpin GmbH, Rheinstetten, Germany), operated using a 30° pulse, a spectral width of 8223.56 Hz, acquisition time of 3.98 s, and a repetition delay of 1 s. Analyses of 1D-spectra were performed with the software ACD/NMR Processor Academic Edition (version 12.01; Advanced Chemistry Development, Inc., Toronto, Canada). Solutes were identified by comparing experimental spectra with reference spectra of known compatible solutes [78]. The chemical shifts of the peaks used for the integration and quantification were 3.27 ppm −N^+^(CH_3_)_3_ for GB and 2.25 ppm −CH_3_ for Ect. At least six culture replicates per salinity were measured and results are given as the mean ± SD/cell volume. Quantification of compatible solutes was done by integrating the peak areas relative to the internal standard TMSP. The calculation of the cell volume of *S*. *salinarum* was based on generic SEM pictures generated as described by [111]. Width, length, and height of different individual cells were measured and used to calculate average values. The cell shape of *S*. *salinarum* was estimated according to [81] with the following formula:

$${\text{V}}_{\text{cylinder}}=\pi *(1/2\;\text{average}\;{\text{width})}^{\text{2}}*\;\text{average}\;\text{length}$$

$${\text{V}}_{2}{}_{\;\text{cones}}=2*(1/3*\pi *(1/2\;\text{average}\;{\text{width})}^{2}*\;\text{average}\;\text{length})$$

The measured amount of intracellular compatible solute per cell was set in relation to the calculated cell volume (average cell volume: 8.01 pl) to estimate the intracellular concentration of the compatible solutes.

Statistical analyses were performed in R Studio (version 3.3.1, http://r-project.org) using the basic package. First, the different data sets were tested for normal distribution using the Shapiro-Wilk test. Second, the Bartlett test for homogeneity of variance was used to test if the variances of the different data sets have been homogenous or not. Because the data sets have been normally distributed and have shown unequal variances, significant differences for the intracellular concentration of GB and Ect in different salinities were calculated by using Welch’s *t* test. A correlation between the extracellular NaCl concentration and the intracellular concentration of compatible solutes was achieved by using a Pearson product-moment correlation.

### ^1^H-NMR control experiments

To ensure that the starvation step was successful and that possibly remaining food bacteria did not disturb ^1^H-NMR measurements, three control experiments were conducted. SEM of filter-fixed cells prepared for NMR analyses was done to screen for potential bacterial contaminations. SEM followed the protocol of [111]. DAPI (4ʹ,6-Diamidin-2-phenylindol; Sigma-Aldrich, Taufkirchen, Germany) staining was conducted to visualize remaining ingested bacteria in the starved ciliates as described in [111]. ^1^H-NMR spectra of the flow-through resulting from the filtration step during ^1^H-NMR sample preparation were generated to assess whether the remaining bacteria in the cultivation medium show the same osmolyte NMR peaks as *S*. *salinarum*.

### Compatible solute accumulation experiments

To investigate whether *S*. *salinarum* is able to accumulate compatible solutes from the external medium, cultures, grown at 9% salinity, were incubated with GB and Ect (final concentration: 1 mM; Sigma-Aldrich), respectively. Additionally, 1 mM Ch, a precursor of GB, was added to the medium to reveal a potential GB synthesis pathway in *S*. *salinarum*. During the growth experiments, cell abundances were counted in triplicates after 3, 6, and 9 d. Pure cultures of *S*. *salinarum* served as control.

### ^13^C_2_-Ch uptake experiments

In general, dynamic metabolic fluxes can be examined by the combination of NMR spectroscopy with the introduction of ^13^C-labeled substrates, such as ^13^C_2_-Ch. As the substrate is metabolized, the ^13^C-label is transferred downstream to metabolic products. Studies and reviews on the principles and applications of dynamic studies with NMR spectroscopy are readily available [112–114]. The ^13^C-label turnover can be detected by direct ^13^C-NMR spectroscopy or, as it was done in our study, indirectly by the coupling of the ^13^C-nucleus with protons as detected by ^1^H-NMR. The advantage of ^1^H-NMR spectroscopy is that it detects both the protons attached to ^12^C-nuclei as well as to ^13^C-nuclei. To assess whether *S*. *salinarum* can synthesize GB by the conversion of Ch, cell cultures with a salinity of 9% were starved for three d, salt shocked by increasing the salinity up to 13% and incubated with ^13^C_2_-Ch for another three d at RT (final concentration: 1 mM; Sigma-Aldrich). Afterwards, the ^13^C-label turnover was detected by ^1^H-NMR. The sample preparation and the peak identification followed the protocol described above. Measurements were applied to three culture replicates.

### Ion imaging

Ion imaging was performed with Na^+^- and K^+^-specific fluorescence markers to investigate whether *S*. *salinarum* uses the salt-in strategy to counterbalance osmotic stress. Therefore, cells were concentrated by centrifugation in a pear-shaped ASTM centrifuge tube (Lenz Laborgals GmbH & Co.) for 5 min at 400 g (Roto Silenta III). For Na^+^ imaging, concentrated cells were resuspended in CoroNa Green loading buffer (DMSO mixed with CoroNa Green, AM Sodium Indicator, Molecular Probes, final concentration: 0.5 μM) and incubated in the dark at 37 °C for 10 min. Incubation time was determined by preliminary experiments with incubation times at 10, 15, 20, 25, 30, 35, 40, and 45 min. Because no significant differences in relative fluorescence intensity at the different time points were observed, the minimum incubation time of 10 min was used for further experiments. Cells were washed three times and resuspended in ASW to remove excess fluorescence dyes. For K^+^ imaging, cells were resuspended in Asante Potassium loading buffer (DMSO mixed with Asante Potassium, TEFLABS, final concentration: 0.5 μM) and incubated in the dark at RT for 30 min. Stained cells were attached to poly-L-lysine-coated glass slides and visualized with a Carl Zeiss Axiostar plus epifluorescence microscope equipped with a specific filter set (Abs/Em for CoroNa Green/Na^+^ complex: 492/516 nm; Abs/Em for Asante Potassium/K^+^ complex: 488 to 517/540 nm). Images were taken with a cooled black and white high resolution CCDMicrocam (Intas, Germany) and the QImager software. During Na^+^ imaging and K^+^ imaging, exposure times for each time frame were 500 ms and 700 ms, respectively, the camera settings for all microphotographs were identical. For Na^+^ imaging, pictures were taken at 4, 9, 13, 17, and 21% salinity. Because no increase in relative fluorescence intensity was observed during K^+^ imaging, pictures were only taken at 4, 10, and 21% salinity.

### Quantification of intracellular ion concentrations and statistical analyses

Analysis of fluorescence intensity was done using the open source software FIJI [115]. Average fluorescence intensity (F) of individual cells was determined by defining three areas of the cell by intensity thresholds: background (I_B_), cytoplasm (I_C_), and vacuoles (I_V_), where I was pixel intensity (I_B_ < I_C_ < I_V_) and I_B_ the mean value of four different background regions. The CRF was calculated as

$${\text{CRF}}_{\text{c}}{=\text{I}}_{\text{C}}-\left({\text{A}}_{\text{C}}{\;\text{x}\;\text{I}}_{\text{B}}\right)\text{,}$$

where A_C_ was the cytoplasm area. CRF was normalized to the measured area as

$${\text{CRF}}_{\text{norm}}{=\text{CRF}}_{\text{C}}{\text{/A}}_{\text{C}}\text{.}$$

Measurements were carried out for at least 10 cells per salinity.

Statistical analyses were performed in R Studio (version 3.3.1, http://r-project.org) as described above. Because the data sets have been normally distributed and homogeneity of variance existed, significant differences for the different salinities were calculated by ANOVA. Because ANOVA only informs about overall differences between the tested groups, a post-hoc Tukey-honest significant difference (HSD) test was run to identify which specific groups differed.

### Ion imaging with short-term adapted cells

To test if the observed increase of intracellular Na^+^ concentration with increasing external salinity of long-term adapted *S*. *salinarum* cells is a result of passive ion-influx or rather a product of active regulated uptake (i.e., presence of specific membrane transporters), short-term experiments were conducted. Therefore, a customized poly-L-lysine-coated microscopic slide was used to enable a quick exchange of the medium by gravity flow. Single *S*. *salinarum* cells adapted to a salinity of 4% were loaded with CoroNa Green fluorescence indicator as described above and attached to the customized slide. During the experiment the salinity was stepwise increased from 4 to 6, 8, 10, 12, 14, 16, and 21%. After each increase in salinity the investigated cell was allowed to rest for 5 min. Then, fluorescence intensity was recorded using epifluorescence microscopy and analysed as outlined above. Measurements were applied to three replicate cells.

### Determination of enzymatic activity at high-salt concentrations

To experimentally investigate the properties of *S*. *salinarum* proteins, the two well-conserved proteins MDH and isocitrate ICDH were tested for their activity under different salt and osmolyte conditions and their protein charge in comparison to respective proteins of *E*. *coli*. Sequence information for both proteins were obtained by genome sequencing of S. *salinarums* macronuclear DNA.

#### Cloning

MDH and ICDH encoding genes from *E*. *coli* were amplified by PCR from genomic DNA and cloned into the expression vector pETDuet-1 using the *Eco*RI and *Sal*I or *Bam*HI and *Hind*III restriction sites, respectively. The gene sequences encoding MDH and ICDH from *S*. *salinarum* genome data were identified by comparison with *Homo sapiens* (cytosolic) MDH1 and (cytosolic) IDH1 sequences. The retrieved sequences from the preliminary genomic data of *S*. *salinarum* presented intact telomere sequences at both ends. One intron was identified in each ORF, according to the criteria defined by [116]. Protein sequences from *S*. *lemnae* and *O*. *trifallax* helped to identify splicing sites. For the MDH_*S*.*salinarum*_ gene the splice donor site was AG↓GTAAAAT and the acceptor site TATAG↓ (removing a 32-nucleotide intron), whereas for ICDH_*S*.*salinarum*_ the donor site was AG↓GTAATAT and the acceptor site TTTAG↓ (removing a 35-nucleotide intron). The ORF sequences without introns were translated using the ciliate genetic code with the Expasy translate tool (UAA and UAG code for Gln). The corresponding protein sequences were introduced in the software from ThermoFisher Scientific to order uncloned, chemically synthesized double-stranded linear DNA fragments (GeneArt Strings; ThermoFisher Scientific, Waltham, MA) with optimized codon usage for *E*. *coli*. The DNA fragments harboring the flanking restriction sites *Eco*RI and *Sal*I were cloned into the expression vector pETDuet-1. DNA sequencing confirmed the correctness of all constructs. Nucleotide and protein sequences of ICDH_*S*.*salinarum*_ and MDH_*S*.*salinarum*_ are given in S1 Text.

#### Protein expression and purification

pETDuet1-His-tag-ICDH and pETDuet1-His-tag-MDH from *E*. *coli* and *S*. *salinarum* were transformed into the expression strain *E*. *coli* BL21 (Novagen, Darmstadt, Germany). Single transformants were allowed to grow overnight at 37 °C in LB liquid medium with ampicillin (100 μg/ml) at 250 rpm in a shaker. The next day, a culture of 2000-ml LB liquid medium was inoculated (1% v/v) at 37 °C. When the cell culture reached an optical density of 0.8, the temperature was lowered to 30 °C and induction was started by addition of 1 mM (final concentration) of IPTG (isopropyl β-D-thiogaloctoside). Cells were harvested by centrifugation (9,000 x *g*, 10 min, 4 °C) after 4 h of induction at 30 °C. The cell pellets were washed with lysis buffer (50 mM NaH_2_PO_4_, 300 mM NaCl, and 10 mM imidazole, pH 8.0). After centrifugation (9,000 x *g*, 10 min, 4 °C) the cell pellets were resuspended in lysis buffer and kept at −20 °C until use. Once thawed, the cell pellets were homogenized through a syringe and submitted to two passages in a french press at 4 °C. After centrifugation (50,000 x *g*, 60 min, 4 °C), the resulting supernatant was mixed with 4 ml Ni-NTA resin (Cube Biotech, Monheim am Rhein, Germany) and homogenized for 1 h at 4 °C for binding. The column material was then poured into an empty column and allowed to settle by gravity. After washing with 20 bed volumes of lysis buffer with 20 mM imidazole, pH 8.0, proteins were eluted with 250 mM imidazole in buffer. For removal of imidazole the dehydrogenase containing fractions were subjected to Sephadex G25 PD-10 column chromatography (25 mM Tris, pH 8.0, 150 mM NaCl). For *S*. *salinarum* proteins the salt concentration in all buffers used in the purification was 2 M NaCl. The purified proteins were divided into aliquots, shock-frozen in liquid nitrogen, and stored at −80 °C.

#### Enzyme activity

MDH and ICDH activities were determined in a 1-mL reaction buffer containing 32.5 mM Tris/HCl, pH 7.5, at indicated molar concentrations of NaCl or 2.5 M GB (pH adjusted with concentrated HCl). For MDH, 0.3 mM NADH was used with oxaloacetate at indicated concentrations. For ICDH, 1 mM MgCl_2_, 0.8 mM NADP^+^ (Sigma-Aldrich) was used with DL-isocitrate at indicated concentrations (Sigma-Aldrich). Assays were started by addition of (freshly diluted) purified enzyme and followed by the decrease (MDH) or increase (ICDH) of the absorbance at 340 nm, resulting from the oxidation of NADH to NAD^+^ or reduction of NADP^+^ to NADPH, respectively. The protein concentrations were determined from the absorbance at 280 nm using the calculated extinction coefficient from the amino acid sequence and were confirmed by the microbiuret method using BSA as protein standard.

## Supporting information

S1 Fig Separate addition of authentic GB to cell extracts. ^1^H-NMR spectra of *S*. *salinarum* cells grown in ASW with a salinity of 13% and added authentic GB. No additional peaks occurred in the spectrum after the addition of authentic GB to the samples. ASW, artificial seawater; GB, glycine betaine; ^1^H-NMR, proton nuclear magnetic resonance; ppm, parts per million.
(TIF)

S2 Fig Separate addition of authentic Ect to cell extracts. ^1^H-NMR spectra of *S*. *salinarum* cells grown in ASW with a salinity of 13% and added authentic Ect. No additional peaks occurred in the spectra after the addition of authentic Ect to the samples. ASW, artificial seawater; Ect, ectoine; ^1^H-NMR, proton nuclear magnetic resonance; ppm, parts per million.
(TIF)

S3 Fig Ethanolic cell extract. ^1^H-NMR spectra of GB and Ect in the ethanolic extract of *S*. *salinarum* cells grown in ASW with a salinity of 9%. No additional peaks occurred by using the ethanolic extraction protocol. ASW, artificial seawater; Ect, ectoine; GB, glycine betaine; ^1^H-NMR, proton nuclear magnetic resonance; ppm, parts per million.
(TIF)

S4 Fig ^1^H-NMR control experiments. (A), Comparison of compatible solute spectra of *S*. *salinarum* and food bacteria. The cell extract of food bacteria did not show any osmolyte peaks. (B), Scanning electron micrograph of filter membrane after the culture filtration step in the ^1^H-NMR sample preparation protocol. Only *S*. *salinarum* cells and filter membrane pores are visible. Bar corresponds to 20 μm. (C), Epifluorescence microscopy of *S*. *salinarum* stained with DAPI after three d of starvation. Six macronuclei and the micronucleus are visible, but no food bacteria. Bar corresponds to 100 μm. ^1^H-NMR, proton nuclear magnetic resonance; HOD, hydrogen oxygen deuterium; ppm, parts per million.
(TIF)

S5 Fig Osmotic stress-dependent accumulation of GB (A) and Ect (B) in *S*. *salinarum*. Based on Welch’s *t* tests, four and two significant changes in intracellular GB and Ect concentrations, respectively, could be revealed. Significance levels based on Welch’s *t* test: 0.01 < *p* ≤ 0.05 ≙ *; 0.001 ˂ *p* ≤ 0.01 ≙ **; *p* ≤ 0.001 ≙ ***. Raw data for this figure can be found in S1 Data. Ect, ectoine; GB, glycine betaine.
(TIF)

S6 Fig Ratio of Ect:GB in *S*. *salinarum* along a salt gradient. With increasing external salinity, the intracellular concentration of Ect increases relative to the concentration of GB. Black colored standard deviations are based on Ect; white colored standard deviations are based on GB (*n =* 6). Raw data for this figure are deposited in S1 Data. Ect, ectoine; GB, glycine betaine.
(TIF)

S7 Fig Comparison of ^1^H-NMR spectra of *S*. *salinarum* incubated with 1,2-^13^C_2_-Ch (A), authentic GB (B) and authentic 1,2-^13^C_2_-GB (C), A proportion of the exogenously provided 1,2-^13^C_2_-Ch was internalized and converted to 1,2-^13^C_2_-GB (A), The doublet peaks of 1,2-^13^C_2_-GB are due to the ^13^C-H splitting from the ^13^C label of C(1) and C(2) in the molecule. C atoms in the chemical structures of GB are color coded. The peak assignment and the chemical shifts are given in the legends of the reference spectra. C, carbon; Ch, choline; Ect, ectoine; GB, glycine betaine; ^1^H-NMR, proton nuclear magnetic resonance; ppm, parts per million.
(TIF)

S8 Fig Relative intracellular sodium concentrations in short-time adapted *S*. *salinarum* cells. The relative Na^+^ concentration was measured in the cytoplasm of single *S*. *salinarum* cells (*n* = 3) while increasing the salinity. After each salinity increase cells were allowed to rest either for 5 min (A) or for 10 min (B). No significant changes in Na^+^ concentrations in the cytoplasm could be observed. Raw data underlying the figures can be found in S1 Data. CRF_norm_, normalized corrected relative fluorescence intensity; Na^+^, sodium.
(TIF)

S9 Fig Phylogenetic tree of malate dehydrogenase. Analysis is based on sequence datasets by [44]. Clustal omega (http://www.ebi.ac.uk/Tools/msa/clustalo/) and iTOL [117] tools were used to render images. iTOL, Interactive Tree Of Life.
(TIF)

S10 Fig Phylogenetic tree of isocitrate dehydrogenase. Analysis is based on sequence datasets by [106]. Clustal omega (http://www.ebi.ac.uk/Tools/msa/clustalo/) and iTOL [117] tools were used to render images. iTOL, Interactive Tree Of Life.
(TIF)

S11 Fig SDS-PAGE of purified N-terminally His-tagged proteins after overexpression in *E*. *coli*. Proteins from *E*. *coli*: (A) MDH, (B) ICDH; from *S*. *salinarum*: (C) MDH, (D) ICDH. Proteins were stained with Coomassie dye. AI, after induction; BI, before induction; E, eluate; FT, flow-through; ICDH, isocitrate dehydrogenase; MDH, malate dehydrogenase; P, pellet; SDS-PAGE, sodium dodecyl sulfatepolyacrylamide gel electrophoresis; SN, supernatant; W, wash.
(TIF)

S1 Table Overview of bacteria included in *S*. *salinarum* cultures and potentially serving as food source. (XLSX)

S1 Text Nucleotide and protein sequences of ICDH_*S*.*salinarum*_ and MDH_*S*.*salinarum*_. ICDH, isocitrate dehydrogenase; MDH, malate dehydrogenase. (DOCX)

S1 Data Excel file containing raw data for Figs 2–6, S5, S6 and S8 Figs. (XLSX)

## Abbreviations

^1^H: hydrogen-1
ASA: aspartate semialdehyde
ASW: artificial seawater
BADH: betaine aldehyde dehydrogenase
c(Ect): intracellular ectoine concentration
c(GB): intracellular glycine betaine concentration
CDH: choline dehydrogenase
Ch: choline
Cl: chloride
CMO: choline monooxygenase
COX: choline oxidase
CRF: corrected relative fluorescence intensity
Ect: ectoine
GB: glycine betaine
GSMT: glycine sarcosine methyltransferase
HSD: honest significant difference
ICDH: isocitrate dehydrogenase
IPTG: isopropyl β-D-thiogaloctoside
K: potassium
kDA: kilodalton
L-glu: L-glutamate
MDH: malate dehydrogenase
Mg: magnesium
Na: sodium
NAD^+^: nicotinamide adenine dinucleotide
NADP^+^: nicotinamide adenine dinucleotide phosphate
*NHX1*: Na+/H+ exchanger
Ni-NTA: nickel-nitrilotriacetic acid
NMR: nuclear magnetic resonance
pI: isoelectric point
ppm: parts per million
RT: room temperature
SAM: S-adenosylmethionine
SDMT: sarcosine dimethylglycine methyltransferase
SDS-PAGE: sodium dodecyl sulfatepolyacrylamide gel electrophoresis
SEM: scanning electron microscopy
TMSP: trimethylsilylpropanoic acid
