# Supplementary figures and images for: Identification of osmoadaptive strategies in the halophile, heterotrophic ciliate Schmidingerothrix salinarum
Source: PLoS Biol. 2018 Jan 22;16(1):e2003892. doi: 10.1371/journal.pbio.2003892 (PMC5794333; doi:10.1371/journal.pbio.2003892)

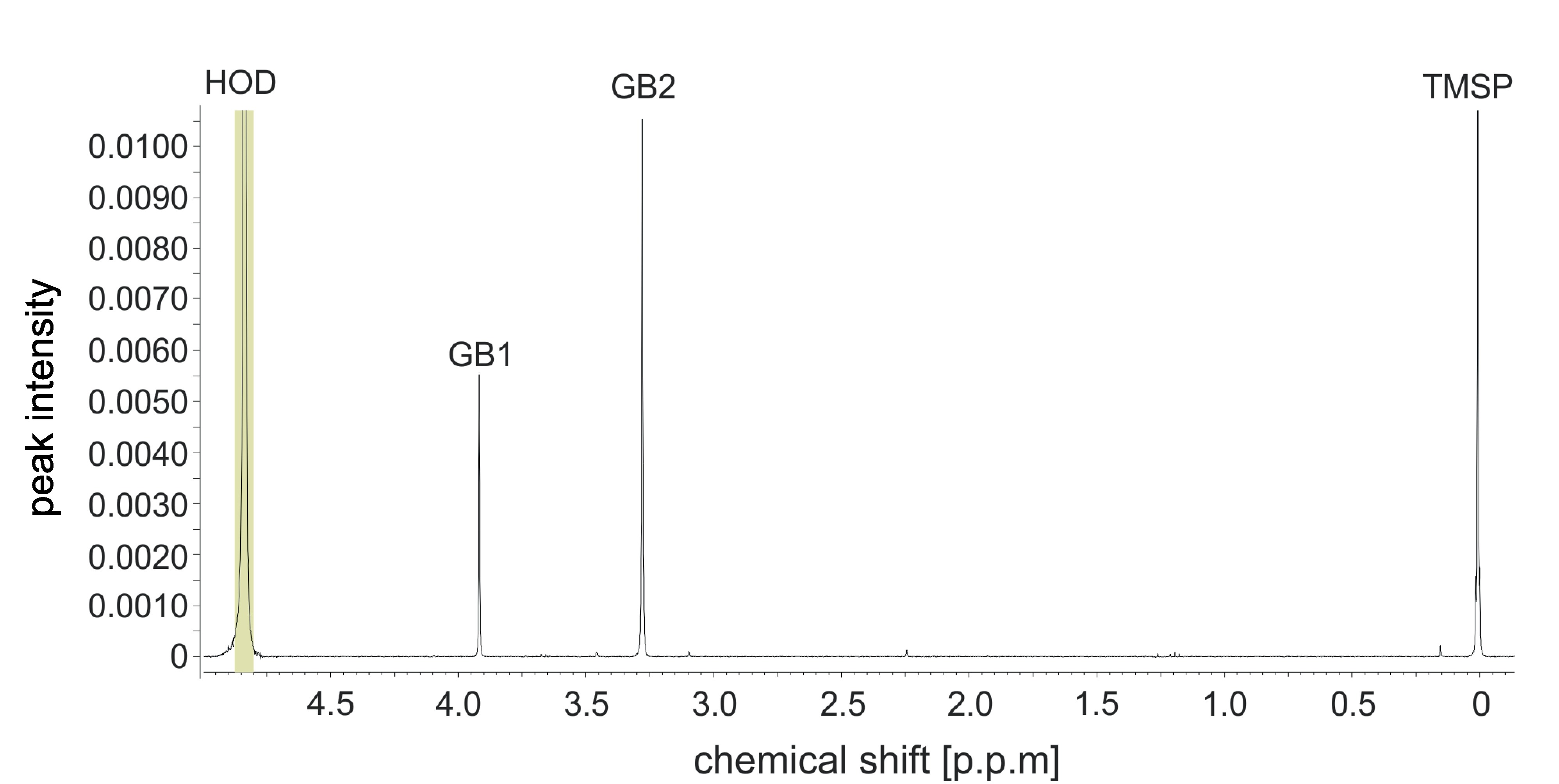

Supplement: S1 Fig — 1H-NMR spectra of S. salinarum cells grown in ASW with a salinity of 13% and added authentic GB. No additional peaks occurred in the spectrum after the addition of authentic GB to the samples. ASW, artificial seawater; GB, glycine betaine; 1H-NMR, proton nuclear magnetic resonance; ppm, parts per million. (TIF) [file pbio.2003892.s001.tif]

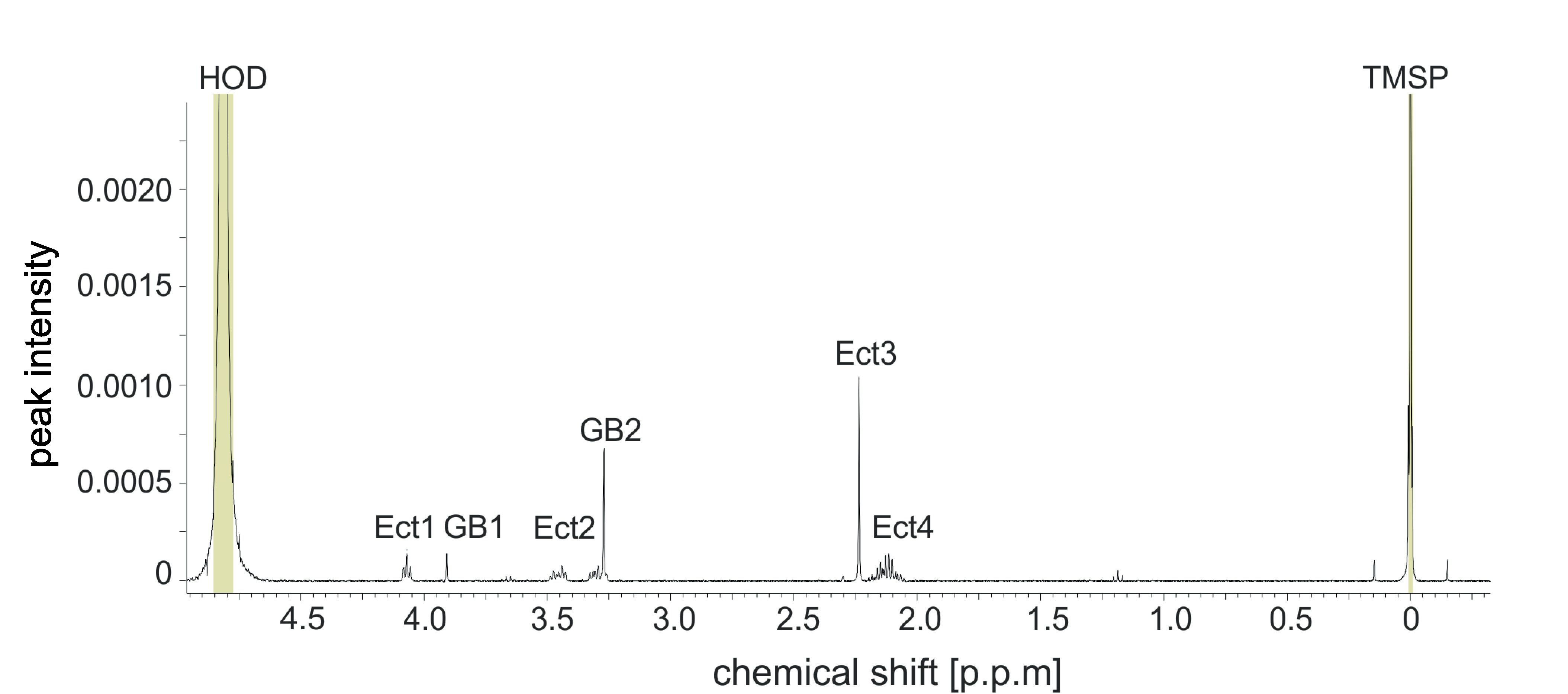

Supplement: S2 Fig — 1H-NMR spectra of S. salinarum cells grown in ASW with a salinity of 13% and added authentic Ect. No additional peaks occurred in the spectra after the addition of authentic Ect to the samples. ASW, artificial seawater; Ect, ectoine; 1H-NMR, proton nuclear magnetic resonance; ppm, parts per million. (TIF) [file pbio.2003892.s002.tif]

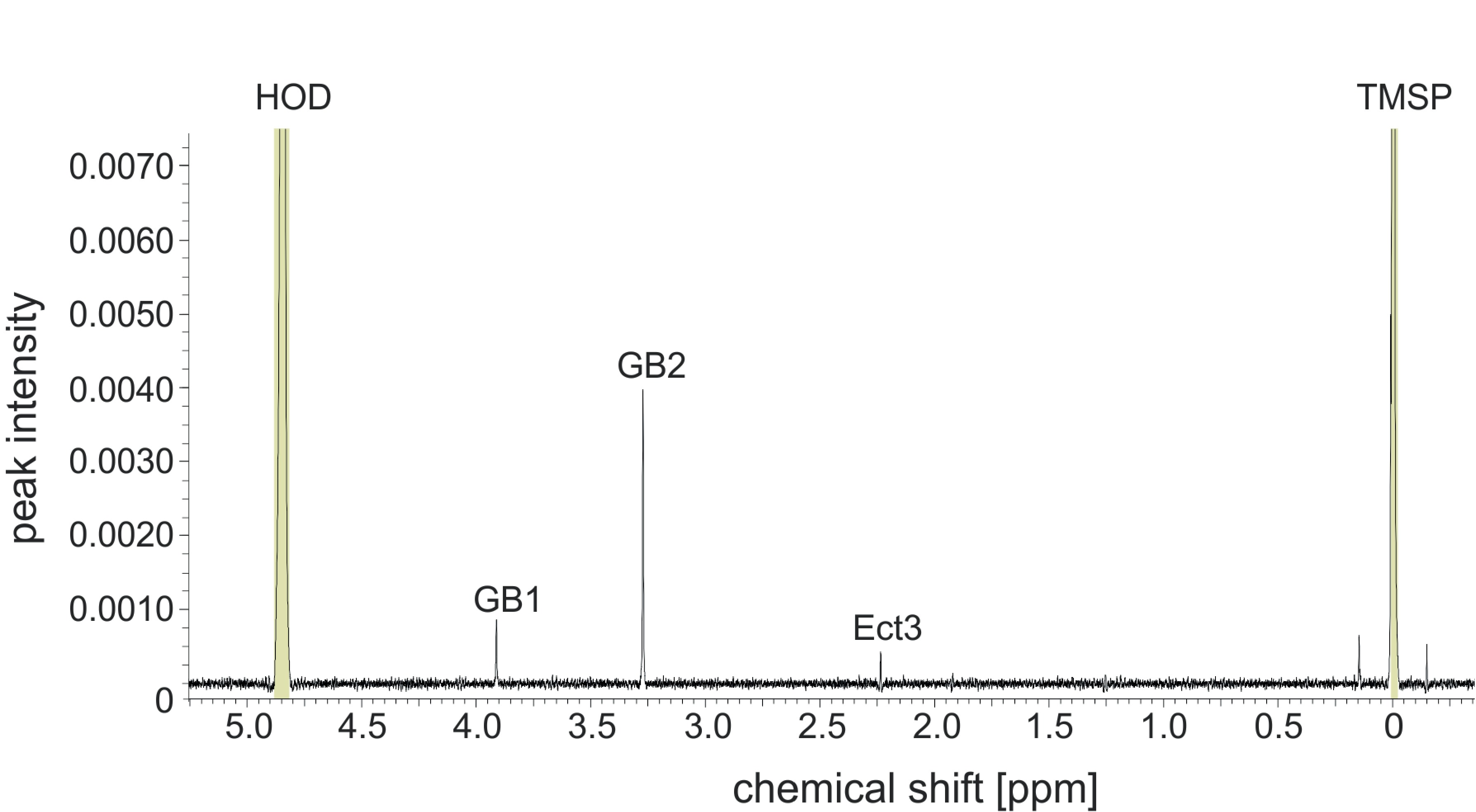

Supplement: S3 Fig — 1H-NMR spectra of GB and Ect in the ethanolic extract of S. salinarum cells grown in ASW with a salinity of 9%. No additional peaks occurred by using the ethanolic extraction protocol. ASW, artificial seawater; Ect, ectoine; GB, glycine betaine; 1H-NMR, proton nuclear magnetic resonance; ppm, parts per million. (TIF) [file pbio.2003892.s003.tif]

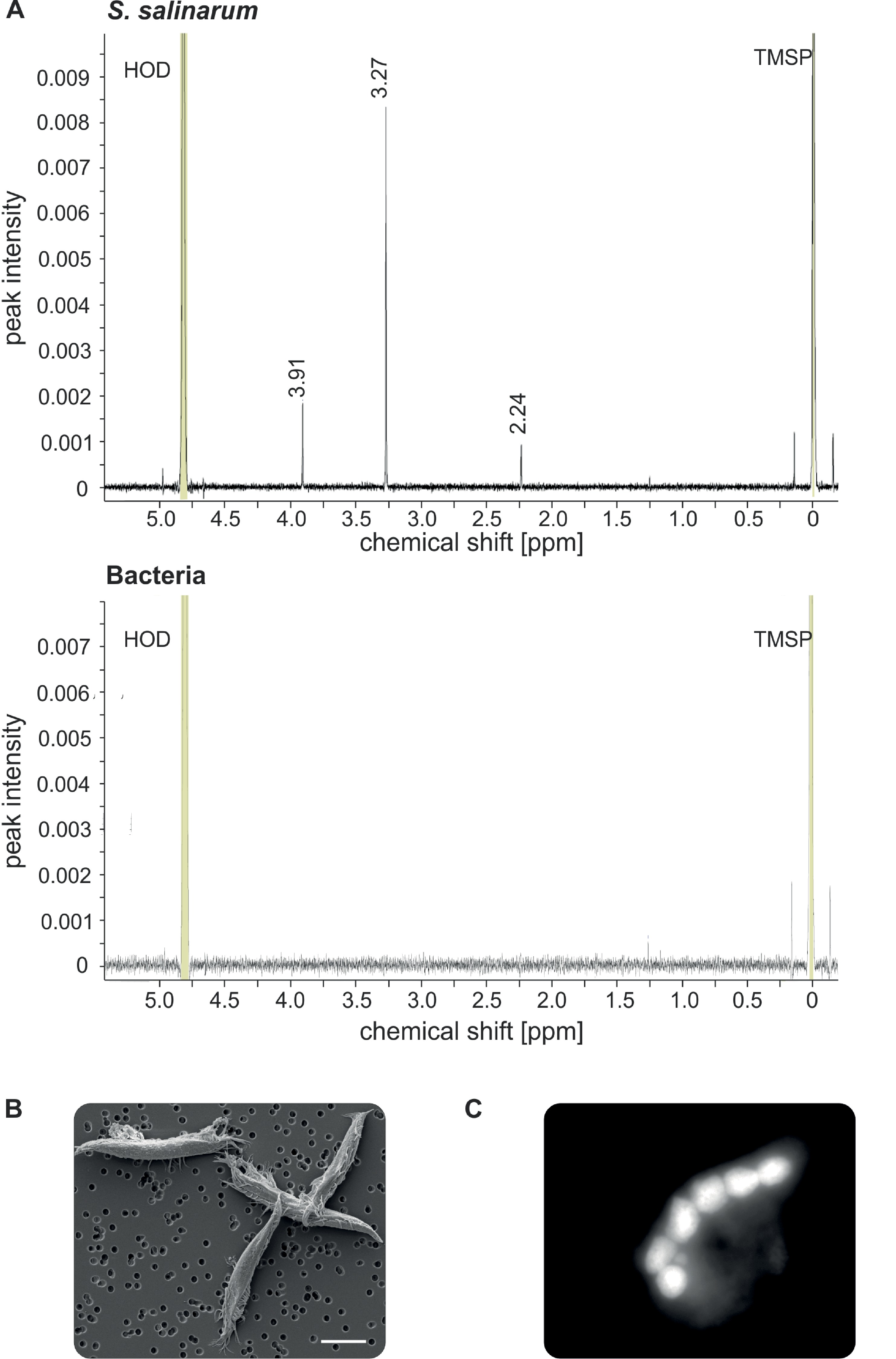

Supplement: S4 Fig — (A), Comparison of compatible solute spectra of S. salinarum and food bacteria. The cell extract of food bacteria did not show any osmolyte peaks. (B), Scanning electron micrograph of filter membrane after the culture filtration step in the 1H-NMR sample preparation protocol. Only S. salinarum cells and filter membrane pores are visible. Bar corresponds to 20 μm. (C), Epifluorescence microscopy of S. salinarum stained with DAPI after three d of starvation. Six macronuclei and the micronucleus are visible, but no food bacteria. Bar corresponds to 100 μm. 1H-NMR, proton nuclear magnetic resonance; HOD, hydrogen oxygen deuterium; ppm, parts per million. (TIF) [file pbio.2003892.s004.tif]

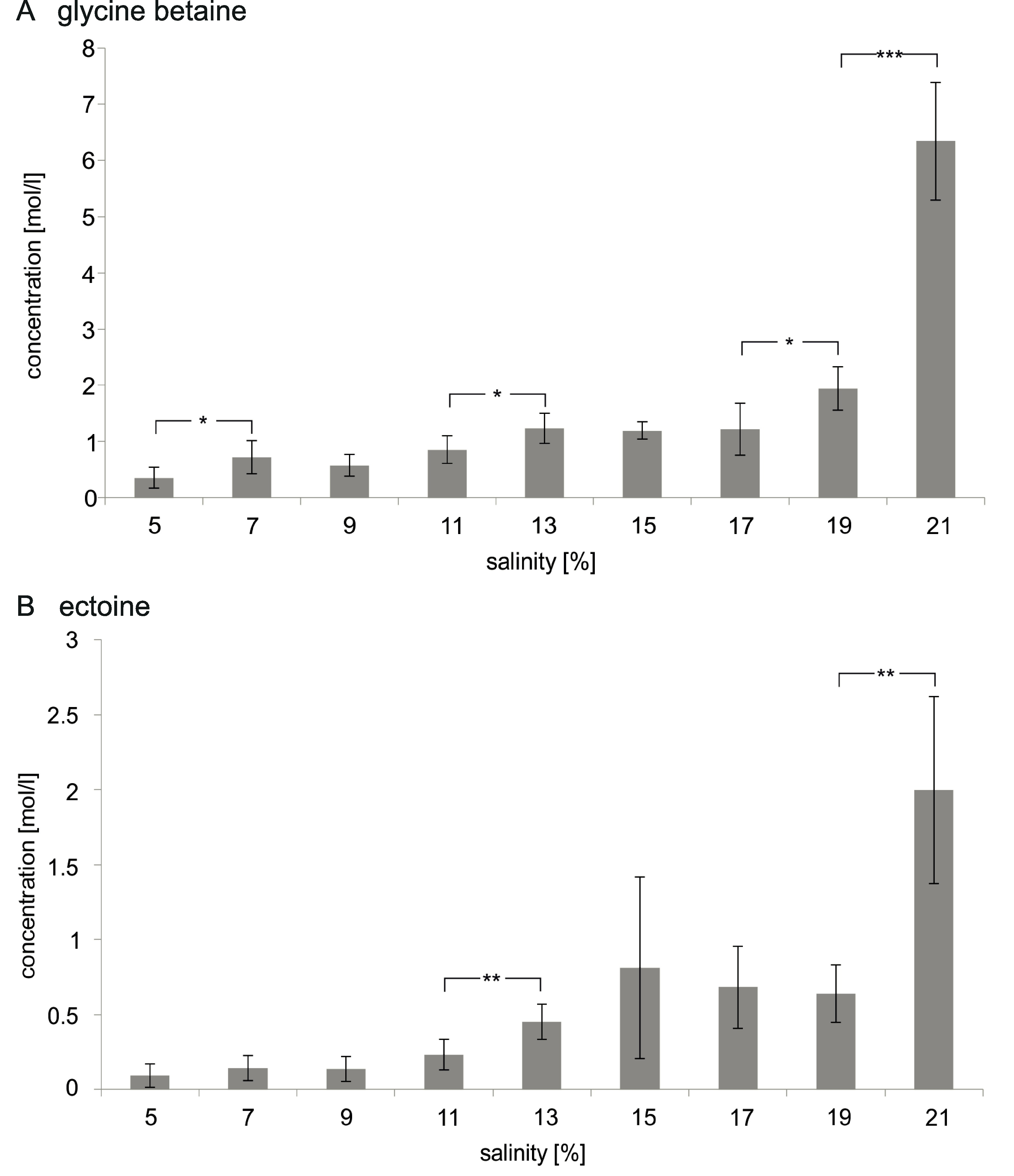

Supplement: S5 Fig — Based on Welch’s t tests, four and two significant changes in intracellular GB and Ect concentrations, respectively, could be revealed. Significance levels based on Welch’s t test: 0.01 < p ≤ 0.05 ≙ *; 0.001 ˂ p ≤ 0.01 ≙ **; p ≤ 0.001 ≙ ***. Raw data for this figure can be found in S1 Data. Ect, ectoine; GB, glycine betaine. (TIF) [file pbio.2003892.s005.tif]

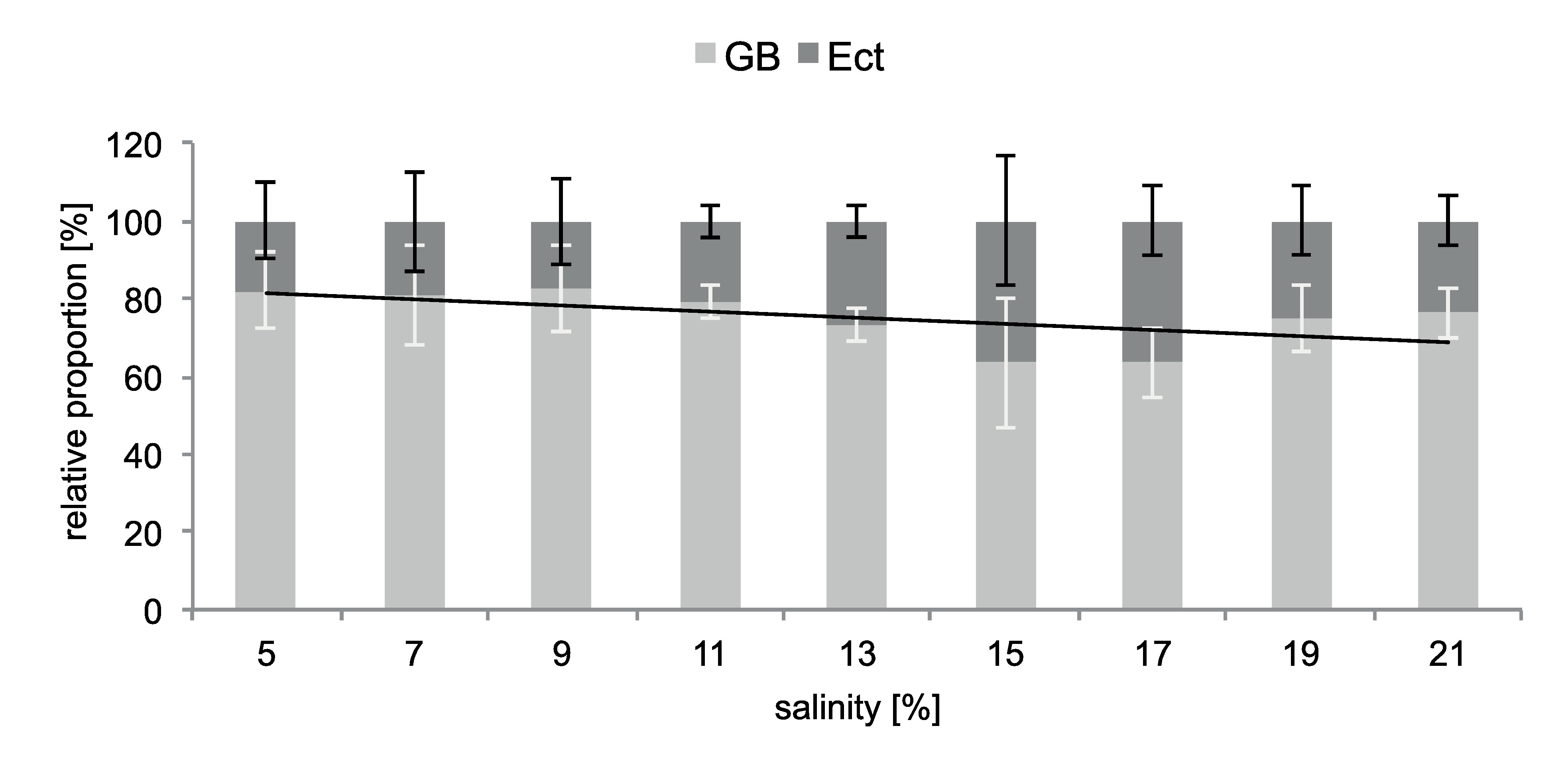

Supplement: S6 Fig — With increasing external salinity, the intracellular concentration of Ect increases relative to the concentration of GB. Black colored standard deviations are based on Ect; white colored standard deviations are based on GB (n = 6). Raw data for this figure are deposited in S1 Data. Ect, ectoine; GB, glycine betaine. (TIF) [file pbio.2003892.s006.tif]

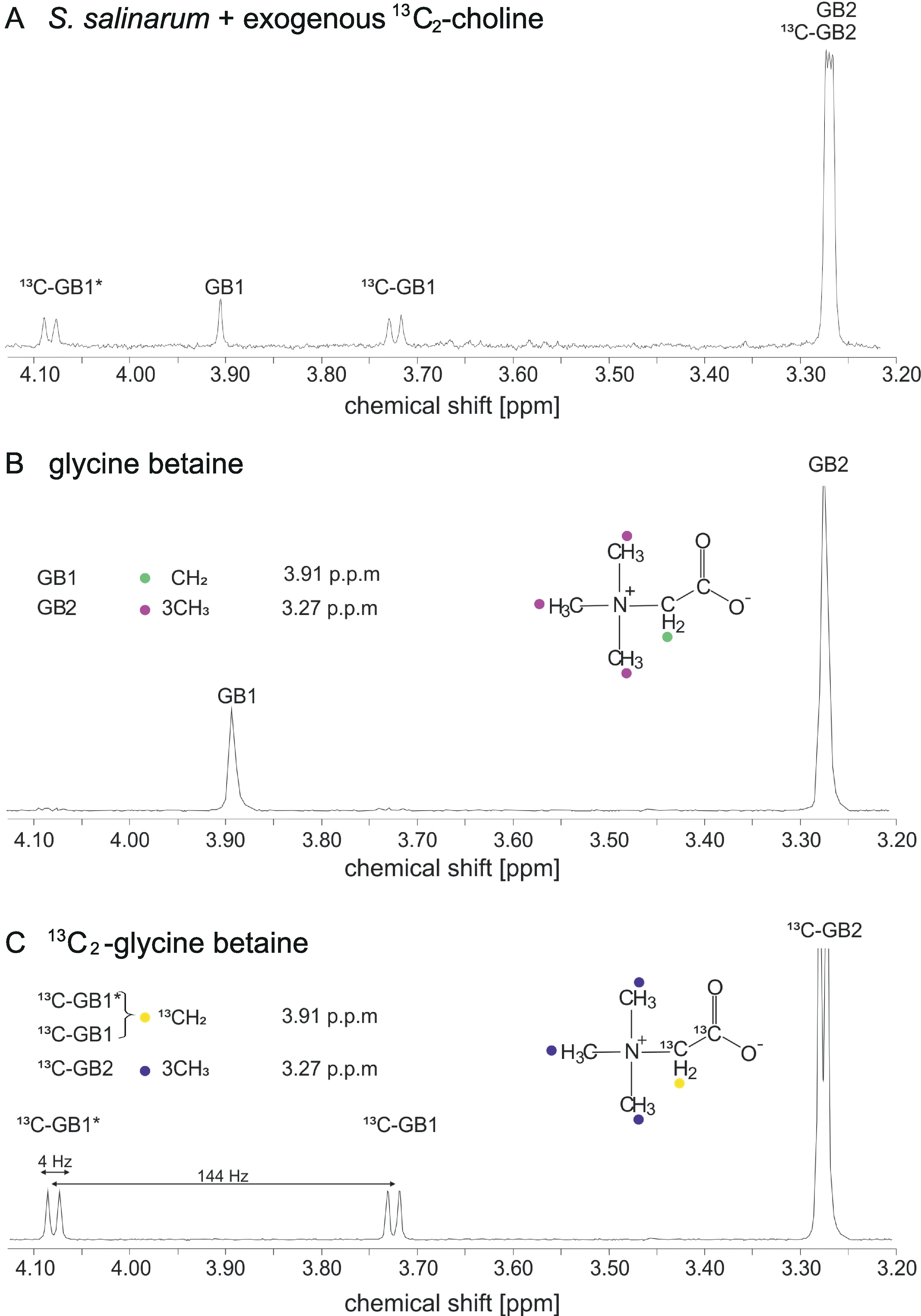

Supplement: S7 Fig — C atoms in the chemical structures of GB are color coded. The peak assignment and the chemical shifts are given in the legends of the reference spectra. C, carbon; Ch, choline; Ect, ectoine; GB, glycine betaine; 1H-NMR, proton nuclear magnetic resonance; ppm, parts per million. (TIF) [file pbio.2003892.s007.tif]

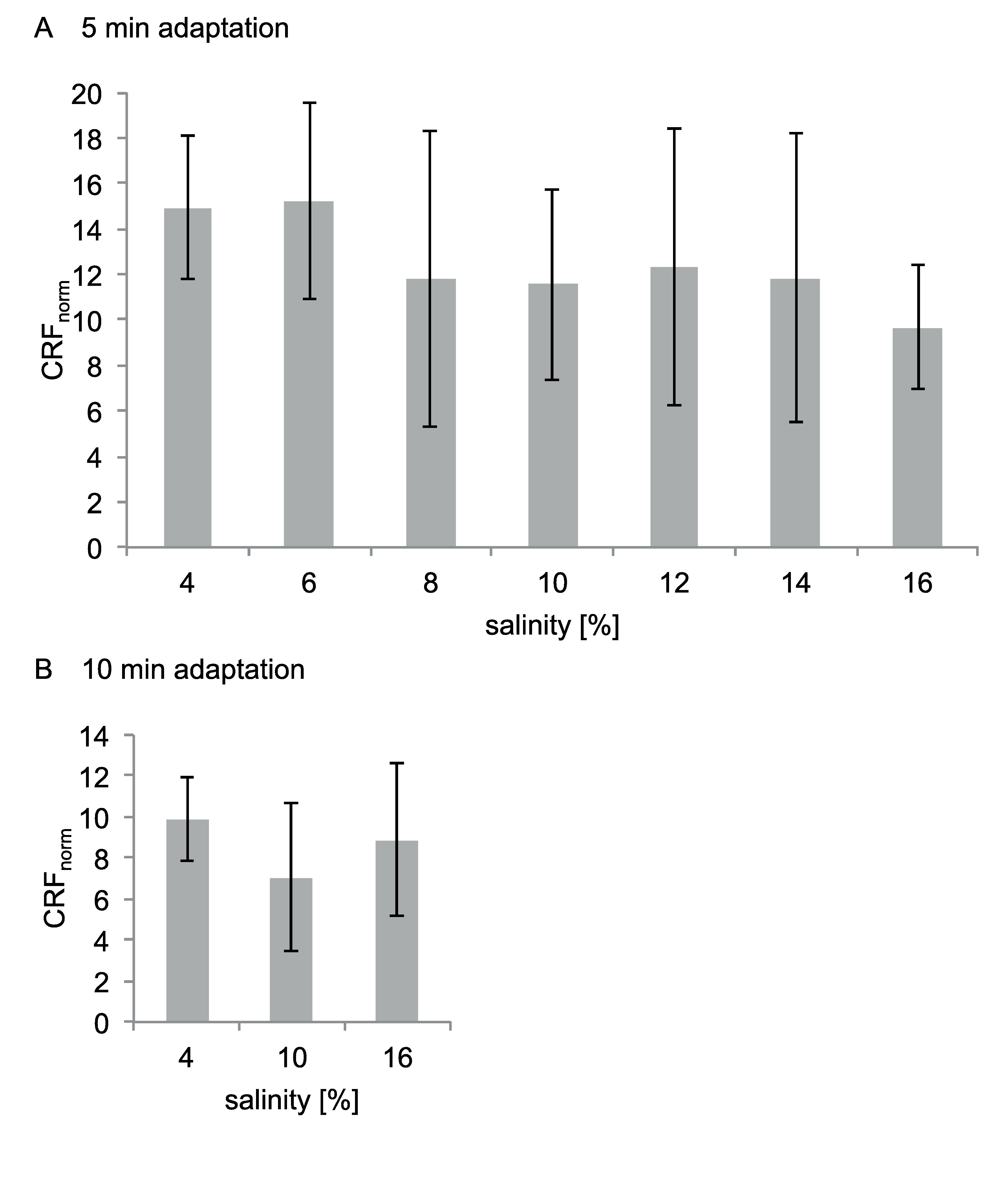

Supplement: S8 Fig — The relative Na+ concentration was measured in the cytoplasm of single S. salinarum cells (n = 3) while increasing the salinity. After each salinity increase cells were allowed to rest either for 5 min (A) or for 10 min (B). No significant changes in Na+ concentrations in the cytoplasm could be observed. Raw data underlying the figures can be found in S1 Data. CRFnorm, normalized corrected relative fluorescence intensity; Na+, sodium. (TIF) [file pbio.2003892.s008.tif]

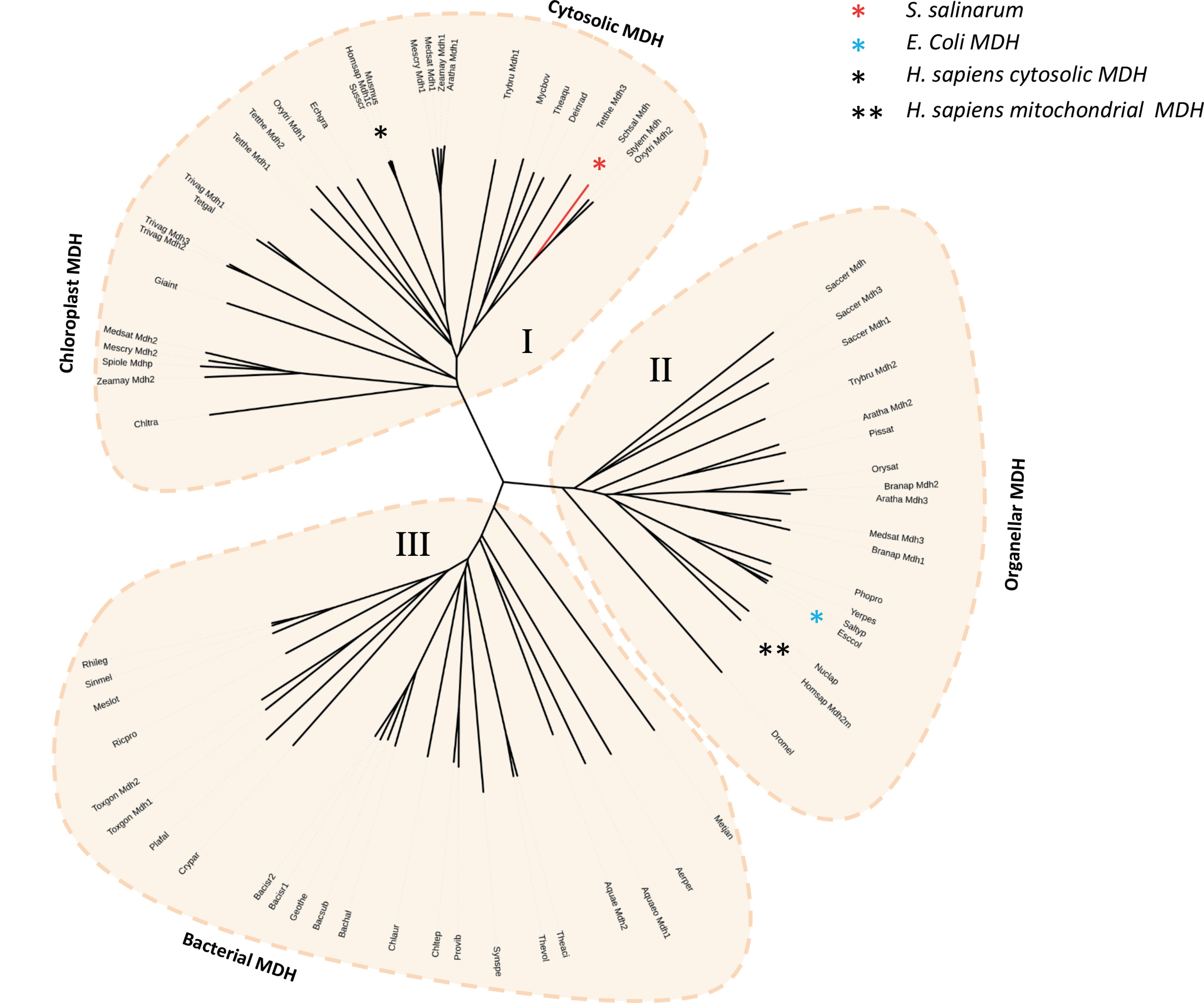

Supplement: S9 Fig — Analysis is based on sequence datasets by [44]. Clustal omega (http://www.ebi.ac.uk/Tools/msa/clustalo/) and iTOL [117] tools were used to render images. iTOL, Interactive Tree Of Life. (TIF) [file pbio.2003892.s009.tif]

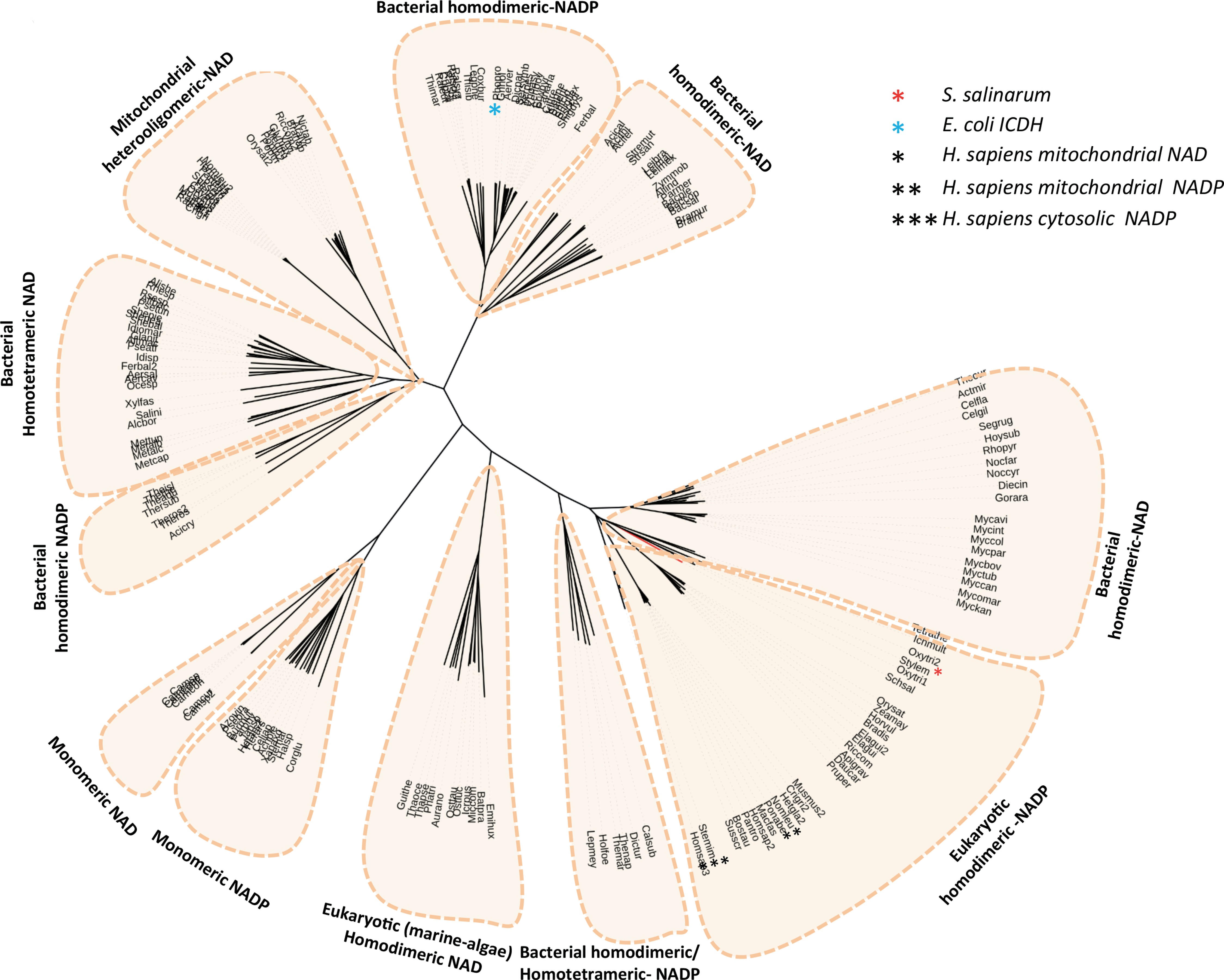

Supplement: S10 Fig — Analysis is based on sequence datasets by [106]. Clustal omega (http://www.ebi.ac.uk/Tools/msa/clustalo/) and iTOL [117] tools were used to render images. iTOL, Interactive Tree Of Life. (TIF) [file pbio.2003892.s010.tif]

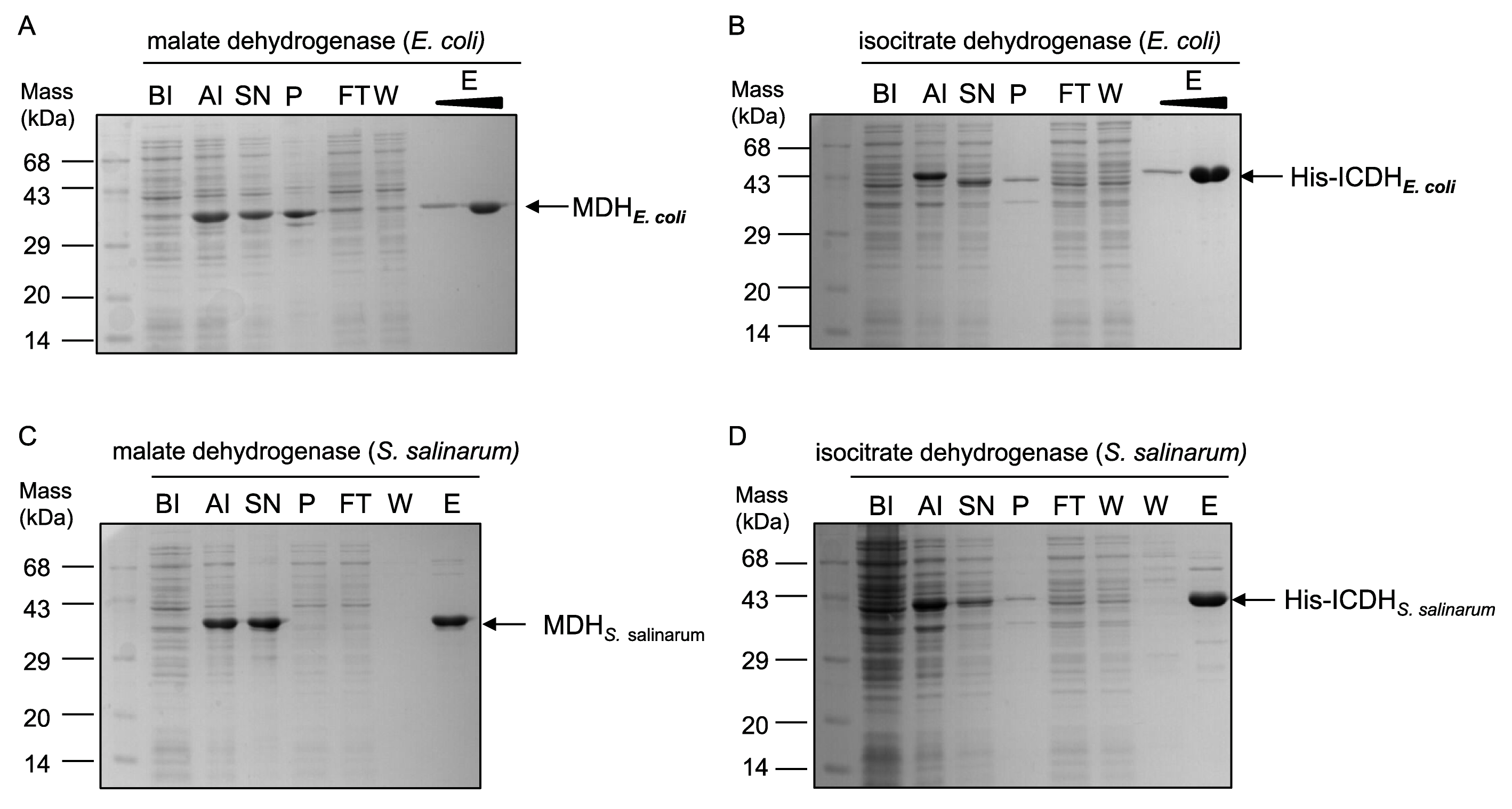

Supplement: S11 Fig — Proteins from E. coli: (A) MDH, (B) ICDH; from S. salinarum: (C) MDH, (D) ICDH. Proteins were stained with Coomassie dye. AI, after induction; BI, before induction; E, eluate; FT, flow-through; ICDH, isocitrate dehydrogenase; MDH, malate dehydrogenase; P, pellet; SDS-PAGE, sodium dodecyl sulfatepolyacrylamide gel electrophoresis; SN, supernatant; W, wash. (TIF) [file pbio.2003892.s011.tif]
