# Supplementary material for: Identification of osmoadaptive strategies in the halophile, heterotrophic ciliate Schmidingerothrix salinarum
Source: PLoS Biol. 2018 Jan 22;16(1):e2003892. doi: 10.1371/journal.pbio.2003892 (PMC5794333; doi:10.1371/journal.pbio.2003892)
Supplement: S1 Text — (DOCX) [file pbio.2003892.s013.docx]

**Supplementary File S2**. Nucleotide and protein sequences of ICDH*_S.salinarum_* and MDH*_S.salinarum_.*

Nucleotide sequences

>Ssalinarum_ICDH_genomic

ATGCAAGCAAAAATCAAGGTAGCTAATCCAGTTGCTGAATTAGATGGTGATGAAATGACCAGAATTATCTGGAAATTCATTAAGGAAAAGCTCATCTTACCATACTTAGATATAAATATTTAATACTTTGATCTTGGGTTAGAAAATAGAGATGCCACAAATGATTAAGTAACTTTTGATGCCGCAAGAGCTATTAAGGAATGCAAAGTAGGAATCAAGTGTGCTACTATCACACCAGACGAGGGAAGAATGACAGAATTCAACTTGAAATAAATGTGGAGGTCTCCAAATGGAACAATCAGAAATGAATTAAATGGAACAGTGTTCAGAGAACCTATCATTATTAAAAACATTCCAAGACTTGTTCCTGGATGGAAAAAACCTATTGTCATTGGAAGACATGCATATGGTGATCAATATAAAGCAACTGATTATGTCGTTGATAAGCCAGGAAAGTTTGAGATCAAATTCACACCAACTGATGGAAGTGAAGTAAAAAGCTTTGAAGTATTTGACTTCAAAGATAAGGGTGTTCTTATGGGAATGTACAACACTGATTCATCAATCAGAGCATTTGCACATGCAAGCTTCAAATATGCTCTTGAAAGAAACTACCCTCTATATATGAGTACTAAGAATACAATATTAAAGAAATATGATGGTAGATTCAAAGATATTTTCGAAGAAATCTACAAGAGTGATTATGAAGAAGAATTCAAAAAGAGAAATCTATGGTATGAACACAGACTTATTGACGATATGGTTGCTTATTGTATTAAGTCAGAAGGTGGATTTGTATGGGCCTGCAAGAACTATGATGGTGACGTATAGTCAGATATTGTAGCTTAAGGATATGGATCACTTGGATTAATGACTAGTGTACTCCTCAATCCCGACGGATGTGTAGAGGCTGAAGCAGCTCACGGAACAGTTACAAGACATTACAGACTCCATCAATAAGGAAAAGAGACCTCTACTAACCCAATTGCATCCATTTTTGCTTGGACTAGAGGATTAGCAGAGAGAGCTAAATTTGACAATAATGATTAATTACTTGCTTTCTCTTAAACTTTAGAGTAAGTGGTTATAGAAACTGTAGAGTAAGGACACATGACAAAGGATTTAGCTATCTGTGTTGCAGGAACCAACAACGTAGGAAAGGATTCTTATCTCAATACATAGGAATTCTTAGACAAGATCAACGAGAATCTAAAGAAAAAGTATCAAGCTTGA

>Ssalinarum_MDH_genomic

ATGCTCTCAAAATCTCTTAGATAACTTAAGCAATTGACACCAGTAACCTAAAGGGCTATGGCCACTAGCTCTGGAGAACAACCTAAAAGAATTGTCGTAACTGGAGGTGCCGGTCAAATTGCATATTCAATTCTTTTCAGACTCGCTAGTGGAGAATTCTTAGGAAAAGATCAAAGAGTAATTCTACATTTGTTAGATCTACCAAACATGGAATAATCTTTGTAAGGAGTAAGAGCTGAATTACATGATTGCGCTTTCCCTCTTCTTGATGATGTCGTTATCACAAGTGACCTCTCCTAAGCCTTTAAAGATGTAGACTATGCTTATCTTGTTGGAGCTAAACCAAGAAGCCAAGGTATGGAAAGAGCTGATCTGTTAAAGGATAATGGTAAAATCTTTGTTGATGTTGGAAAGGCAATGAATGATAATGCTAAAAGAGATTGCAAAACTATTGTAGTTGGAAATCCAGCCAATACTAATTGCCTTATTACTTAACATTATGCTAAAGATATCCCAGCTGAGAATTTCACTGCAATGACAAGATTAGATCATAACAGAGCTCTCACATAACTTGCTCTTAAAACAGGAACATCAGTTACTGACATTAAGTAATTAGCCGTTTGGGGAAATCACTCACCAACAATGTACCCAGATATTAGATATGCAACTATTAAGGGAAAGAAAGCTACCGACTTAGTTGACTAGAAATGGGTCAATGAAGAATTCACCCCAAGAGTTTAAAAAAGAGGAGCTGAGATTATTAATCTGAGAAAACTCTCTAGTGCTGCTTCAGCTGGTAATGCTGCTATTGACCATATGAGAGACTGGGTTAAAGGAAGTAATGAATGGTAAAGCATTGCATTTAAATCTGATGGAAAGCTCTACAATATTCCAGAAGGATTAATCTTCTCAGTACCATGCACAACTGCAAATGGAAAATACAAGCCAATTGAAGGACTTTCACTTGATGACGAAGACAGTCAATAGAAAATCAAGAAGACTACTGACGAATTATTAAATGAAAGAAAGGAAGTTGAATTCTTGTTAAAATGA

Protein sequences

>Ssalinarum_His-tagged_ICDH

MGSSHHHHHHSQDPNS

MQAKIKVANPVAELDGDEMTRIIWKFIKEKLILPYLDINIQYFDLGLENRDATNDQVTFDAARAIKECKVGIKCATITPDEGRMTEFNLKQMWRSPNGTIRNELNGTVFREPIIIKNIPRLVPGWKKPIVIGRHAYGDQYKATDYVVDKPGKFEIKFTPTDGSEVKSFEVFDFKDKGVLMGMYNTDSSIRAFAHASFKYALERNYPLYMSTKNTILKKYDGRFKDIFEEIYKSDYEEEFKKRNLWYEHRLIDDMVAYCIKSEGGFVWACKNYDGDVQSDIVAQGYGSLGLMTSVLLNPDGCVEAEAAHGTVTRHYRLHQQGKETSTNPIASIFAWTRGLAERAKFDNNDQLLAFSQTLEQVVIETVEQGHMTKDLAICVAGTNNVGKDSYLNTQEFLDKINENLKKKYQA

>Ssalinarum_His-tagged_MDH

MGSSHHHHHHSQDPNS

MLSKSLRQLKQLTPVTQRAMATSSGEQPKRIVVTGGAGQIAYSILFRLASGEFLGKDQRVILHLLDLPNMEQSLQGVRAELHDCAFPLLDDVVITSDLSQAFKDVDYAYLVGAKPRSQGMERADLLKDNGKIFVDVGKAMNDNAKRDCKTIVVGNPANTNCLITQHYAKDIPAENFTAMTRLDHNRALTQLALKTGTSVTDIKQLAVWGNHSPTMYPDIRYATIKGKKATDLVDQKWVNEEFTPRVQKRGAEIINLRKLSSAASAGNAAIDHMRDWVKGSNEWQSIAFKSDGKLYNIPEGLIFSVPCTTANGKYKPIEGLSLDDEDSQQKIKKTTDELLNERKEVEFLLK
